# Identifying pleiotropic variants and candidate genes for fertility and reproduction traits in Holstein cattle via association studies based on imputed whole-genome sequence genotypes

**DOI:** 10.1186/s12864-022-08555-z

**Published:** 2022-04-28

**Authors:** Shi-Yi Chen, Flavio S. Schenkel, Ana L. P. Melo, Hinayah R. Oliveira, Victor B. Pedrosa, Andre C. Araujo, Melkaye G. Melka, Luiz F. Brito

**Affiliations:** 1 Department of Animal Sciences, Purdue University, 270 S. Russell Street, West Lafayette, IN 47907-2041 USA; 2 Farm Animal Genetic Resources Exploration and Innovation Key Laboratory of Sichuan Province, Sichuan Agricultural University, Chengdu, 611130 Sichuan China; 3 Centre for Genetic Improvement of Livestock, Department of Animal Biosciences, University of Guelph, Guelph, ON N1G 2W1 Canada; 4 Department of Reproduction and Animal Evaluation, Rural Federal University of Rio de Janeiro, Seropédica, RJ 23897-000 Brazil; 5 Department of Animal Sciences, State University of Ponta Grossa, Ponta Grossa, PR 84030-900 Brazil; 6 Department of Animal and Food Science, University of Wisconsin River Falls, River Falls, WI 54022 USA

**Keywords:** Cattle breeding, Genome-wide association study, GWAS, QTL, Whole-genome sequence variants

## Abstract

**Background:**

Genetic progress for fertility and reproduction traits in dairy cattle has been limited due to the low heritability of most indicator traits. Moreover, most of the quantitative trait loci (QTL) and candidate genes associated with these traits remain unknown. In this study, we used 5.6 million imputed DNA sequence variants (single nucleotide polymorphisms, SNPs) for genome-wide association studies (GWAS) of 18 fertility and reproduction traits in Holstein cattle. Aiming to identify pleiotropic variants and increase detection power, multiple-trait analyses were performed using a method to efficiently combine the estimated SNP effects of single-trait GWAS based on a chi-square statistic.

**Results:**

There were 87, 72, and 84 significant SNPs identified for heifer, cow, and sire traits, respectively, which showed a wide and distinct distribution across the genome, suggesting that they have relatively distinct polygenic nature. The biological functions of immune response and fatty acid metabolism were significantly enriched for the 184 and 124 positional candidate genes identified for heifer and cow traits, respectively. No known biological function was significantly enriched for the 147 positional candidate genes found for sire traits. The most important chromosomes that had three or more significant QTL identified are BTA22 and BTA23 for heifer traits, BTA8 and BTA17 for cow traits, and BTA4, BTA7, BTA17, BTA22, BTA25, and BTA28 for sire traits. Several novel and biologically important positional candidate genes were strongly suggested for heifer (*SOD2*, *WTAP*, *DLEC1*, *PFKFB4*, *TRIM27*, *HECW1*, *DNAH17*, and *ADAM3A*), cow (*ANXA1*, *PCSK5*, *SPESP1*, and *JMJD1C*), and sire (*ELMO1*, *CFAP70*, *SOX30*, *DGCR8*, *SEPTIN14*, *PAPOLB*, *JMJD1C*, and *NELL2*) traits.

**Conclusions:**

These findings contribute to better understand the underlying biological mechanisms of fertility and reproduction traits measured in heifers, cows, and sires, which may contribute to improve genomic evaluation for these traits in dairy cattle.

**Supplementary Information:**

The online version contains supplementary material available at 10.1186/s12864-022-08555-z.

## Background

Fertility, reproduction, calving, and fertility disorders represent a group of traits that directly impact the economic efficiency and animal welfare in the dairy industry [1, 2]. However, the long-term genetic selection for production traits (especially milk yield) in Holstein cattle has negatively impacted fertility and reproductive performance due to their unfavorable genetic correlations [3–5]. Fertility and reproduction traits were only added to worldwide official genetic evaluations over the past two decades [6, 7]. In this context, the more recent implementation of genomic selection (GS) in North American Holstein cattle has greatly contributed to improving genetic gains for fertility and reproduction traits [8]. The usually low heritability estimates [9] and great complexity of biological mechanisms affecting fertility and reproductive performance impact GS accuracy, which relies, among other factors, on population-specific linkage disequilibrium (LD) between genotype markers and causal variants. Therefore, a promising approach is to genotype and use the causal or tightly linked variants within known quantitative trait loci (QTL) related to fertility and reproduction traits in GS schemes, which is expected to improve the prediction accuracy for such traits [10]. Despite the great efforts that have been made to identify QTL, functional genes, and putative causal variants related to fertility and reproduction traits in dairy cattle, it is expected that many potential candidate variants still remain to be uncovered, especially with the increase in detection power achieved by using whole-genome sequence (WGS) variants.

Genome-wide association studies (GWAS) have been popularly performed as a standard method for QTL mapping and candidate gene discovery in both humans and other species [11]. For instance, Fortes et al. [12] and Ma et al. [3], based on a systematic review, reported that a considerable number of QTL and candidate genes located in the *Bos taurus* autosomes (BTA) and in the X chromosome are associated with fertility and reproduction traits. However, most of these studies used the low- or medium-density single-nucleotide polymorphism (SNP) panels, which might hinder the discovery of candidate genes and causal variants. Due to the reduced cost of high-throughput sequencing approaches, several large-scale genome resequencing initiatives have been undertaken, such as the 1,000 Bull Genomes Project [13] and the Canada’s Ten Thousand Cows Genome Project [14]. Based on these large reference populations, WGS variants can be obtained cost-effectively through computational imputation from SNP panels to WGS [15, 16]. The use of WGS variants for GWAS can enhance the discovery of variant-trait associations especially when there is only short-range LD surrounding causal variants [17]. Previous studies have successfully used real or imputed WGS variants for performing GWAS of fertility and reproduction traits in other dairy cattle populations (e.g., [18–20]).

There is a plethora of traditional and novel trait definitions for measuring fertility and reproductive performance in females and males, which are further complicated by alternative definitions across breeding programs [3, 21, 22]. Furthermore, fertility and reproduction traits recorded in heifers and lactating cows have always been treated as different traits, i.e., they have been analyzed separately due to their usually low genetic correlations [9, 23]. Therefore, the detection of pleiotropic variants affecting multiple fertility and reproduction traits is paramount for unraveling their biological background. In this context, Bolormaa et al. [24] proposed an efficient multiple-trait analysis for directly combining the estimated effects of SNPs from single-trait GWAS based on a chi-square statistic, which facilitates the discovery of potential pleiotropic variants. In dairy cattle, this method has been applied to multiple fertility and reproduction traits, which were mainly based on SNP array data (e.g., [25–27]).

In this study, we first performed single-trait GWAS analyses for 18 fertility and reproduction traits in North American Holstein cattle, using imputed WGS genotypes. Following the method proposed by Bolormaa et al. [24], the multiple-trait chi-square statistics were subsequently calculated regarding different trait categories, including heifer traits, cows traits, sire traits, and their combinations. Our main objective was to identify candidate pleiotropic SNPs and genes associated with various fertility and reproduction traits in heifers, cows, and sires, which will contribute to a better understanding of the underlying biological mechanisms of fertility and reproduction traits in North American Holstein cattle.

## Results

### Deregressed breeding values and imputed SNP variants

The analyzed traits and their phenotypic summaries (i.e., deregressed estimated breeding values, dEBV) are shown in Table 1. The number of included animals that have phenotypes ranged from 3,803 for SCSc (sire calf survival – cow) to 5,986 for CA (calving ability). The average accuracy of dEBV (± standard deviation, SD) was 0.55 ± 0.02. After performing the quality control (QC), about 5.6 million SNPs (ranging from 5,396,362 for CA to 5,880,012 for SCSc) were retained for single-trait GWAS analyses. These SNPs were distributed across all autosomes with a mean (± SD) pairwise distance of 445 ± 1,478 bp and a mean (± SD) minor allele frequency (MAF) of 18.7 ± 15.6% (Table S1).

**Table 1:** Traits, summary statistics, and trait categories of multiple-trait analysis

**Traits**	**Full names of traits**	**dEBV**^a^				**Accuracy**		**Trait categories used for multiple-trait analysis**^b^				
		N	Mean	Min	Max	Mean	SD	**H**	**C**	**S**	**HC**	**HCS**
AFS	Age at first service	5,123	81	-1,790	1,992	0.56	0.02	√			√	√
FSTCh	First service to conception heifer	5,451	114	-1,934	2,134	0.55	0.03	√			√	√
NRRh	Non-return rate heifer	5,374	86	-1,939	2,137	0.55	0.03	√			√	√
CEh	Calving ease heifer	4,623	122	-1,726	1,925	0.56	0.02	√			√	√
CSh	Calf survival heifer	5,168	121	-1,887	2,082	0.55	0.03	√			√	√
CA	Calving ability	5,986	110	-1,881	2,074	0.56	0.02	√	√		√	√
CTFS	Calving to first service	4,800	125	-1,847	2,046	0.56	0.02		√		√	√
DO	Days open	5,034	93	-1,773	1,970	0.55	0.02		√		√	√
FSTCc	First service to conception cow	5,036	88	-1,835	2,032	0.55	0.02		√		√	√
NRRc	Non-return rate cow	5,283	105	-1,923	2,118	0.55	0.02		√		√	√
CEc	Calving ease cow	4,761	101	-1,814	2,012	0.56	0.02		√		√	√
CSc	Calf survival cow	5,045	122	-1,956	2,156	0.55	0.03		√		√	√
DCA	Daughter calving ability	5,191	116	-1,874	2,072	0.55	0.02		√		√	√
DF	Daughter fertility	5,529	106	-1,851	2,048	0.55	0.02		√		√	√
SCEh	Sire calving ease heifer	5,782	85	-1,974	2,167	0.56	0.02			√		√
SCEc	Sire calving ease cow	5,133	112	-1,974	2,171	0.56	0.02			√		√
SCSh	Sire calf survival heifer	4,687	109	-1,897	2,103	0.54	0.02			√		√
SCSc	Sire calf survival cow	3,803	113	-1,887	2,100	0.53	0.02			√		√

^a^dEBV: deregressed estimated breeding values

^b^The trait categories are defined with respective to heifers (H), cows (C), sires (S), heifers and cows (HC), and all the animals together (HCS), respectively

### Summaries of single-trait and multiple-trait GWAS analyses

**Fig. 1:**
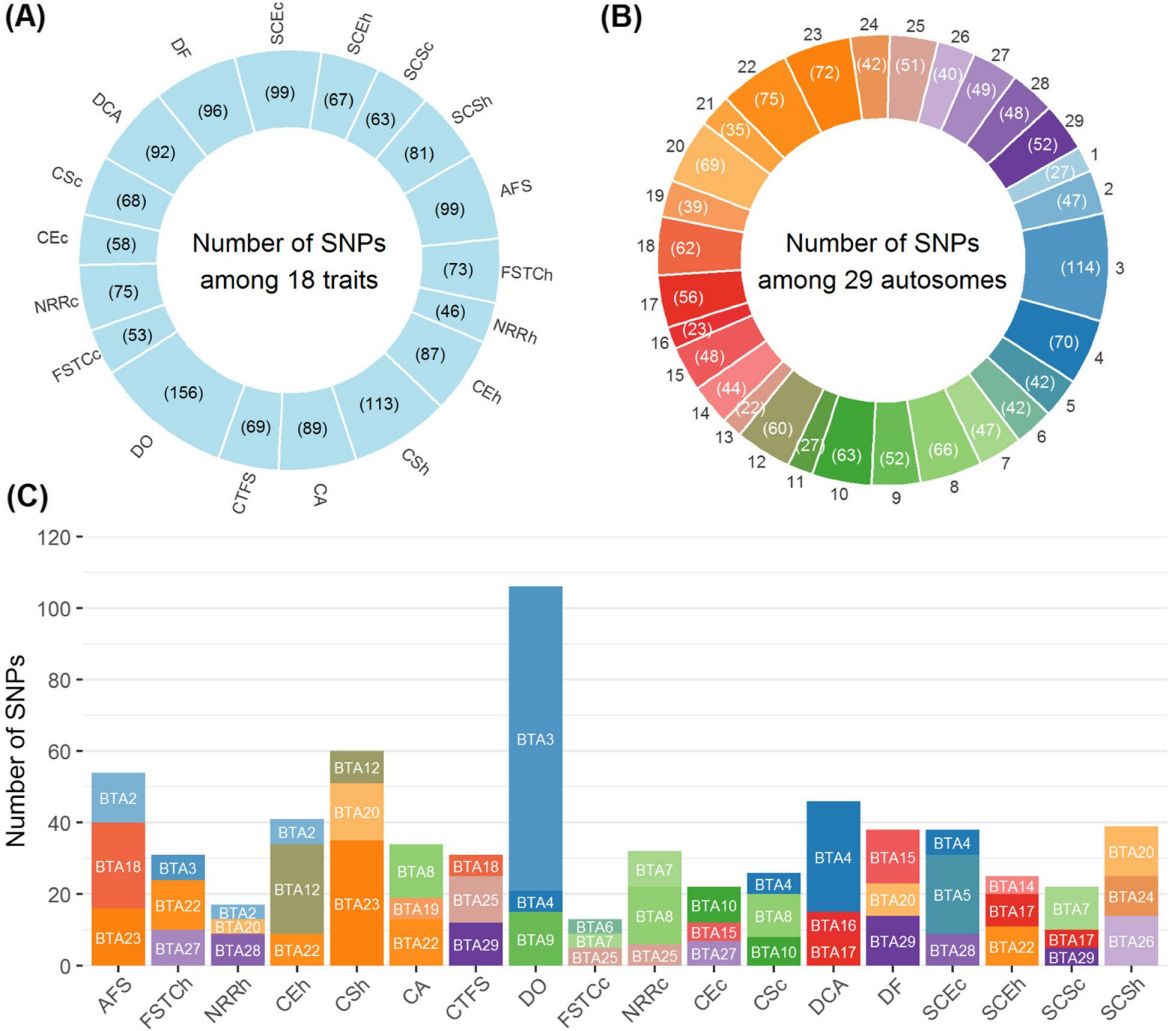
Significant SNPs revealed by single-trait GWAS. Beside the numbers of SNPs identified among 18 fertility and reproduction traits (**A**) and 29 autosomes (**B**), the top three chromosomes with the largest numbers of significant SNPs were shown for each trait (**C**). AFS = Age at first service; FSTCh = First service to conception heifer; NRRh = Non-return rate heifer; CEh = Calving ease heifer; CSh = Calf survival heifer; CA = Calving ability; CTFS = Calving to first service; DO = Days open; FSTCc = First service to conception cow; NRRc = Non-return rate cow; CEc = Calving ease cow; CSc = Calf survival cow; DCA = Daughter calving ability; DF = Daughter fertility; SCEh = Sire calving ease heifer; SCEc = Sire calving ease cow; SCSh = Sire calf survival heifer; SCSc = Sire calf survival cow

The single-trait GWAS revealed a total of 1,484 SNPs that were significantly associated with the 18 fertility and reproduction traits with no overlapping SNPs between traits. The highest and lowest numbers of significant SNPs were observed for DO (days open) and NRRh (non-return rate heifer), respectively (Fig. 1A). These significant SNPs were broadly distributed across all autosomes (Fig. 1B), with the greatest number of significant SNPs located on BTA3, BTA22, and BTA23. For each trait, the top three chromosomes with the greatest numbers of significant SNPs are shown in Fig. 1C. BTA4, BTA20, and BTA22 were observed in common for four traits [i.e., CSc (calf survival cow), DCA (daughter calving ability), DO, and SCEc (sire calving ease cow) for BTA4, CSh (calf survival heifer), DF (daughter fertility), NRRh, and SCSh (sire calf survival heifer) for BTA20, and CA, CEh (calving ease heifer), FSTCh (first service to conception heifer), and SCEh (sire calving ease heifer) for BTA22]. The detailed distribution of significant SNPs identified across traits and chromosomes is shown in Table S2. The number of QTL identified in the single-trait GWAS ranged from 38 for NRRh to 73 for SCEc. There were three QTL overlapping between CA and CTFS (calving to first service), and three overlapping between CSh and SCEc (Table S3). The highest estimated genome inflation factor was equal to 1.090 (95% CI of 1.075–1.104) for DCA, and its mean ± SD across all traits was equal to 1.052 ± 0.024 (Fig. S1).

For the multiple-trait analyses, the defined trait categories of heifer, cow, and sire traits, and their combinations are shown in Table 1. There were 425 significant SNPs revealed by multiple-trait analysis, including 87 for heifers, 72 for cows, 84 for sires, 96 for heifers and cows, and 86 for all animals. Of these, only one SNP (BTA9:21,350,462) was shared between heifers and cows, whereas no SNP for sire traits was shared with heifers, cows, or heifers and cows (Fig. S2). When both heifer and cow traits were analyzed together, there were 15 and 25 significant SNPs overlapping with the independent analyses of heifer and cow traits, respectively. In summary, the multiple-trait analyses revealed a total of 328 unique significant SNPs, which are distributed across all autosomes.

### Multiple-trait analysis for heifer traits

**Fig. 2:**
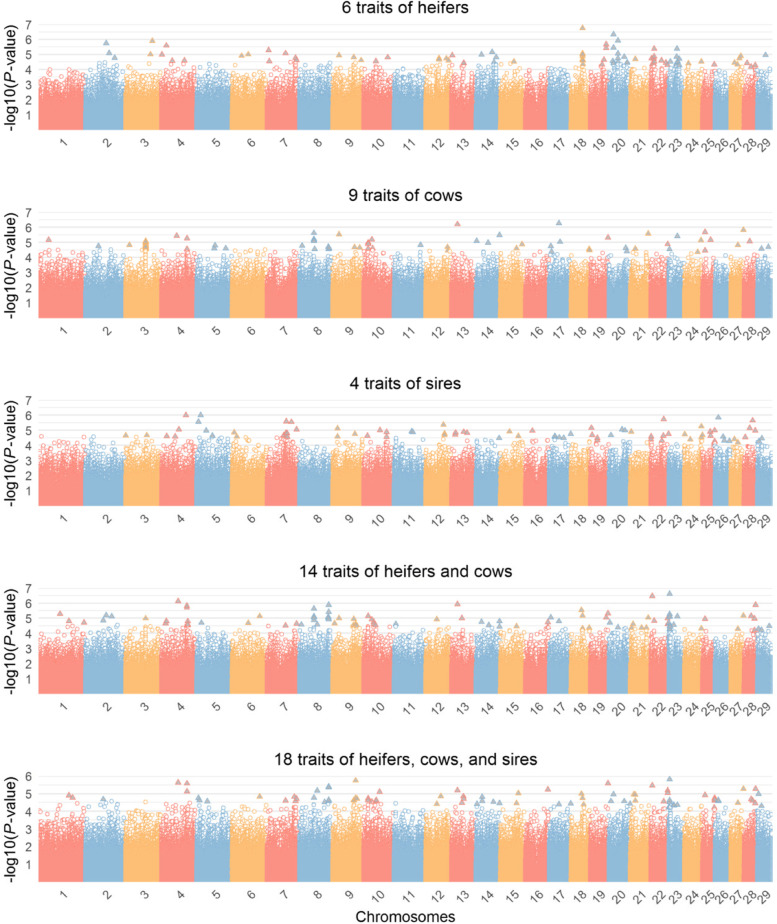
Manhattan plots for multiple-trait analysis for different trait categories. Statistically significant SNP are denoted by triangles

The multiple-trait analysis of six heifer traits revealed 87 significant SNPs broadly distributed across 22 chromosomes (Fig. 2 and Table 2). Of these, the highest numbers of significant SNPs were observed on BTA20, BTA22, BTA23, and BTA18. The three most significant SNPs were located on BTA18, BTA20, and BTA20. A total of 176 protein-encoding and eight long non-coding RNAs (lncRNA) genes were found within ± 100 Kb regions around the significant SNPs, and 41 SNPs were located in the exonic, intronic, or upstream/downstream regions of 36 genes. All 87 significant SNPs were clustered into 69 QTL and three QTL (BTA18:39.58–40.01 Mb, BTA20:15.57–15.94 Mb, and BTA20:29.77–30.06 Mb) were identified based on three or more SNPs. Furthermore, 21 of these QTL (30.4%) overlapped with 138 previously reported reproduction-related QTL in cattle (Table S4). Among them, one QTL on BTA3:89.78–89.98 Mb overlapped with 98 previously reported QTL that are associated with luteal activity (LA) and conception rate (CR).

**Table 2:** Significant SNPs and candidate genes from multiple-trait analysis for six heifer fertility and reproduction traits

**Chr**	**Position** **(bp)**	***P* values**	**Genomic location**	**Candidate genes** **(within ± 100 kb)**^a^	**N of QTL**^b^
BTA2	70,633,250	1.7755e-06	Intergenic	*EN1*	0
	80,773,959	8.2906e-06	Intron	***TMEFF2***	0
	98,894,327	1.703e-05	Intergenic	—	0
BTA3	83,818,837	9.9389e-06	Exon	***PATJ***	0
	89,878,033	1.2513e-06	Intergenic	—	98
BTA4	2,476,516	1.0209e-05	Intergenic	—	0
	17,304,670	2.5726e-06	Intergenic	—	0
	36,768,135	2.6301e-05	Intergenic	ENSBTAG00000053333*	0
	77,892,378	2.4934e-05	Intergenic	*HECW1*	0
BTA6	31,807,526	1.248e-05	Intergenic	—	2
	54,108,303	9.9442e-06	Intergenic	—	1
BTA7	6,177,026	5.1964e-06	Intron	*CPAMD8*, ***F2RL3***, *SIN3B*, *NWD1*	1
	8,193,333	2.7732e-05	Exon	*CASP14*, *CCDC105*, ***SLC1A6***, ENSBTAG00000053903, *OR7C19*, *OR7A85*, ENSBTAG00000051946	0
	62,865,717	8.3985e-06	Intergenic	*FAT2*, *SPARC*, ENSBTAG00000054940, *ATOX1*	0
	97,438,333	1.6934e-05	Intergenic	—	0
	100,189,059	2.344e-05	Intergenic	*ST8SIA4*	2
BTA9	21,350,462	1.1343e-05	Intergenic	—	0
	71,191,104	1.5112e-05	Intergenic	—	0
	95,975,870	2.4538e-05	Intergenic	ENSBTAG00000052316, SOD2, WTAP, ACAT2, *TCP1*, *MRPL18*, *PNLDC1*	5
BTA10	41,323,422	2.7906e-05	Intergenic	—	0
	81,606,975	1.5652e-05	Intron	*SLC10A1*, ***SMOC1***	1
BTA12	45,686,110	1.8064e-05	Intergenic	—	0
	45,712,972	2.0906e-05	Intergenic		
	69,768,461	1.8444e-05	Intergenic	—	0
	77,682,412	2.495e-05	Intron	***NALCN***	4
BTA13	3,327,860	1.1246e-05	Intergenic	*ANKEF1*	0
	42,023,779	3.7745e-05	Downstream	***NXT1***, *GZF1*, ***NAPB***, ENSBTAG00000027420, *MGC133636*, *CST8*, ENSBTAG00000015949	0
	42,049,979	3.7745e-05	Intron		
BTA14	18,883,604	1.0029e-05	Intergenic	—	3
	54,326,109	7.0163e-06	Intergenic	—	1
	61,221,242	3.2749e-05	Intergenic	*CTHRC1*, *FZD6*, *BAALC*	3
	67,859,596	1.5109e-05	Intergenic	*PTDSS1*	0
BTA15	45,999,990	2.9729e-05	Intergenic	*OR6A2*, *OR6B18*, ENSBTAG00000027525, *OR6B17*, ENSBTAG00000037603, *OR2D4*, *OR2D3G*, *OR2AG1E*, *OR2AG1G*, *OR2AG1*, *OR2AG2*	0
BTA18	39,676,071	1.3142e-05	Intron	*ZNF19*, *ZNF23*, ENSBTAG00000053994*, ***CALB2***, ***CMTR2***, **ENSBTAG00000051512**	0
	39,750,065	2.1267e-05	Intergenic		
	39,767,356	8.5276e-06	Exon		
	39,797,494	1.8113e-07	Downstream		
	39,854,578	9.5369e-06	Upstream		
	39,905,828	2.4327e-05	Intron		
	41,105,349	4.5111e-05	Intron	***ZNF536***	2
	41,112,039	2.5866e-05	Intron		
BTA19	39,685,286	4.588e-05	Exon	*PLXDC1*, ***CACNB1***, *RPL19*, ENSBTAG00000050597, *STAC2*, ENSBTAG00000008368, *FBXL20*	0
	46,856,509	2.2533e-05	Intron	***EFCAB3***, *METTL2A*, *TLK2*	2
	53,776,030	3.7096e-06	Intron	***DNAH17***, *PGS1*, *SOCS3*	0
	53,779,550	2.1366e-06	Intron		
BTA20	12,849,264	2.7392e-05	Intron	**ENSBTAG00000049964**	0
	15,673,813	3.5073e-06	Intergenic	*HTR1A*	0
	15,788,934	4.5398e-07	Intergenic		
	15,839,035	3.8454e-05	Intergenic		
	29,868,266	8.7288e-06	Intergenic	ENSBTAG00000054476	0
	29,957,655	1.2013e-06	Intergenic		
	29,957,766	3.1125e-05	Intergenic		
	29,959,061	1.8094e-05	Intergenic		
	51,156,345	1.4415e-05	Intron	***CDH12***	0
	62,043,263	3.4353e-05	Intron	***CTNND2***	0
	62,043,442	3.5997e-05	Intron		
BTA21	17,250,280	2.0376e-05	Intron	***AGBL1***	0
BTA22	2,072,527	1.8504e-05	Intergenic	*EOMES*	0
	5,159,344	4.422e-05	Intron	***TGFBR2***, *GADL1*	1
	9,601,560	4.4335e-05	Intergenic	*ARPP21*	0
	9,634,144	1.8949e-05	Intergenic		
	11,393,650	1.341e-05	Intron	***CTDSPL***, *VILL*, *PLCD1*, *DLEC1*	0
	11,701,385	2.9746e-05	Intron	*ACAA1*, ENSBTAG00000054286, *MYD88*, ***OXSR1***, ENSBTAG00000050531*, *SLC22A14*, *XYLB*, *ACVR2B*	0
	11,737,948	4.1052e-06	Downstream		
	35,777,156	2.4904e-05	Intron	***MAGI1***	0
	51,337,027	3.512e-05	Intron	*CELSR3*, *SLC26A6*, *TMEM89*, *UQCRC1*, ENSBTAG00000049917*, ENSBTAG00000052088*, ENSBTAG00000049028*, *UCN2*, ***PFKFB4***, *SHISA5*, ENSBTAG00000048942, *TREX1*, *ATRIP*, *TMA7*, *CCDC51*	2
	55,900,928	4.9063e-05	Intergenic	*TAMM41*, *TRH*, *TMCC1*	1
BTA23	5,726,221	2.9329e-05	Intergenic	—	0
	25,235,958	5.2155e-05	Intron	*GSTA1*, *GSTA5*, *GSTA3*, *GSTA4*, ***CILK1***, *FBXO9*, *GCM1*	0
	25,819,703	4.2745e-05	Intergenic	ENSBTAG00000013919, ENSBTAG00000048364, ENSBTAG00000038397, ENSBTAG00000015565, *BOLA-DRA*, *BTNL2*, ENSBTAG00000034945, ENSBTAG00000007618	0
	26,689,257	1.4214e-05	Intergenic	—	0
	28,036,775	4.1923e-06	Intron	ENSBTAG00000051047, **ENSBTAG00000007075**, ENSBTAG00000053433, *POU5F1*, *TCF19*, *CCHCR1*, *PSORS1C2*, *CDSN*, *C23H6orf15*	1
	28,634,425	3.1365e-05	Intergenic	*BOLA-NC1*, *JSP.1*, ENSBTAG00000037421, *BOLA*	0
	30,058,286	2.9988e-05	Intron	*ZNF311*, ENSBTAG00000052294*, ***TRIM27***, ENSBTAG00000053198	0
	34,904,897	1.9645e-05	Intergenic	*GHE4*, *PRP2*, ***PRP14***, *PRP9*	0
	34,928,894	5.2141e-05	Intron		
BTA24	13,978,039	3.8859e-05	Intron	***PIK3C3***	0
	58,070,140	2.9176e-05	Intron	*ZNF532*, **ENSBTAG00000022829**, *SEC11C*	0
BTA25	38,933,869	4.7617e-05	Intron	*FBXL18*, ENSBTAG00000050734*, ***TNRC18***, *SLC29A4*, *WIPI2*	3
BTA27	13,585,281	3.5455e-05	Intron	***TENM3***	1
	23,857,214	4.66e-05	Intron	***DLC1***	0
	30,435,395	1.768e-05	Intergenic	—	0
	34,758,502	1.2659e-05	Intron	*ADAM2*, ***ADAM3A***	3
BTA28	10,602,903	3.7999e-05	Intergenic	*RYR2*, *ZP4*	1
	24,802,568	6.4777e-05	Intron	*PBLD*, *HNRNPH3*, ***RUFY2***, *DNA2*, *SLC25A16*	0
	39,062,420	5.1602e-05	Intergenic	*GHITM*, *C10orf99*, *CDHR1*	0
BTA29	27,630,533	1.1083e-05	Intergenic	*OR8G5*, *OR8G47*, *OR8D2*, *OR8B1U*, *OR8B1S*, *OR8B1AE*, ENSBTAG00000051999, *OR8B3G*, ENSBTAG00000050103, *OR8B3*	0

^a^Candidate genes are represented by gene symbol when available, otherwise by the Ensembl gene ID. The long noncoding RNA genes are marked by asterisk (*). The genes directly linked to SNPs are further denoted in bold

^b^The number of known and reproduction-associated QTL found in Cattle QTL Database v43 (www.animalgenome.org)

The functional analyses revealed that the identified 184 candidate genes were significantly enriched into 21 Gene Ontology (GO) terms and Kyoto Encyclopedia of Genes and Genomes (KEGG) pathways (Fig. S3). Among them, the immunity-associated biological functions were predominant, such as the antigen processing and presentation that was associated with multiple genes within the regions of BTA23:25.72–25.92 Mb and BTA23:28.53–25.73 Mb. These genes included the major histocompatibility complex (MHC), class II, DR alpha (*BOLA-DRA*), MHC class I antigen (*BOLA-NC1*), MHC class I heavy chain (*BOLA*), and MHC Class I JSP.1 (*JSP.1*). Another significantly enriched GO term, the “prolactin receptor binding”, was found related to three genes, named growth hormone E4 (*GHE4*), prolactin-related protein 14 (*PRP14*), and prolactin-related protein IX (*PRP9*), within the region of BTA23:34.80–35.03 Mb.

### Multiple-trait analysis for cow traits

There were 72 SNPs significantly associated with cow traits (Fig. 2 and Table 3). These significant SNPs were distributed across 25 chromosomes, with the highest number of SNPs observed on BTA3, BTA8, and BTA10. The three most significant SNPs were located on BTA17, BTA13, and BTA27, respectively. There were 37 significant SNPs located within the exonic, intronic, or upstream/downstream regions of 23 genes, while 101 candidate genes were found within ± 100 Kb of 35 intergenic SNPs. Fifty-six QTL were found, and two of them (BTA3:67.59–67.79 Mb, BTA8:49.18–49.38 Mb) were supported by three or more significant SNPs. By searching the Cattle QTL Database, 17 QTL (30.4%) overlapped with 31 previously reported QTL that are associated with calving interval (CI), CR, first service conception (FSC), and number of inseminations per conception (IPC) (Table S5).

**Table 3:** Significant SNPs and candidate genes from multiple-trait analysis for nine cow fertility and reproduction traits

Chr	Position (bp)	*P* values	Genomic location	Candidate genes (within ± 100 kb)^a^	N of QTL^b^
BTA1	34,156,863	6.8092e-06	Intergenic	*CADM2*	0
BTA2	44,192,878	1.7484e-05	Intron	***CACNB4***	0
BTA3	13,060,985	1.5004e-05	Intergenic	ENSBTAG00000053098, ENSBTAG00000052866*, ENSBTAG00000054932, ENSBTAG00000051454	0
	67,686,622	2.6373e-05	Intron	***ST6GALNAC5***	0
	67,687,557	2.2745e-05	Intron		
	67,687,632	1.0234e-05	Intron		
	67,687,904	1.6141e-05	Intron		
	67,688,010	8.0894e-06	Intron		
	67,688,259	1.4877e-05	Intron		
	67,691,020	1.2185e-05	Intron		
	67,691,132	9.1178e-06	Intron		
	67,691,448	2.7514e-05	Intron		
	67,691,487	1.6939e-05	Intron		
	67,691,717	2.6505e-05	Intron		
	67,693,137	1.9264e-05	Intron		
	67,693,371	2.0276e-05	Intron		
BTA4	52,090,769	3.731e-06	Intergenic	*TES*, ENSBTAG00000055251	0
	86,337,952	2.7549e-05	Intron	***PTPRZ1***	0
	86,338,437	5.4702e-06	Intron		
BTA5	59,741,942	2.5619e-05	Downstream	***OR9K2***, *OR9K15*, *OR9K1*, *OR9K2F*, *OR9K1B*	1
	61,923,964	1.5754e-05	Intergenic	None	2
	98,326,111	2.4699e-05	Intergenic	None	0
BTA8	9,613,389	1.6984e-05	Intron	***KIF13B***, ***HMBOX1***	0
	48,903,003	2.4027e-06	Intergenic	ENSBTAG00000048940*, *TMC1*, ENSBTAG00000052698	0
	49,280,626	6.2112e-06	Intergenic	ENSBTAG00000052764*, *ANXA1*	0
	49,282,386	7.11e-06	Intergenic		
	49,282,702	8.1027e-06	Intergenic		
	52,344,611	2.7461e-05	Intron	***PCSK5***	0
	97,982,105	1.9975e-05	Intergenic	None	0
	98,998,669	2.7653e-05	Intron	***PTPN3***	0
	100,989,652	2.3223e-05	Intron	***ECPAS***, *ZNF483*, *PTGR1*, *DNAJC25*, *GNG10*	0
BTA9	21,350,462	2.972e-06	Intergenic	None	0
	73,443,174	2.0733e-05	Intron	***AHI1***, ENSBTAG00000046495	0
	91,316,160	2.1955e-05	Intergenic	ENSBTAG00000020723, ENSBTAG00000049872, *SCAF8*	1
BTA10	15,091,095	1.0356e-05	Intergenic	*PIAS1*, *CALML4*, *CLN6*, *FEM1B*, *ITGA11*	2
	15,499,538	2.5633e-05	Intron	***CORO2B***	0
	15,737,285	1.2939e-05	Intergenic	*ANP32A*, ENSBTAG00000051138*, *SPESP1*	0
	27,718,962	6.5343e-06	Downstream	***OR4F67B***, *OR4G18*, *OR4K36*, *OR4G8*, *OR4G9*, *OR4G2*	0
	30,942,681	2.4868e-05	Intron	***DPH6***	0
BTA11	89,383,411	1.5143e-05	Intergenic	None	3
BTA12	74,138,771	2.0551e-05	Intergenic	*HS6ST3*, ENSBTAG00000045566, *OXGR1*	0
	74,922,982	3.4154e-05	Intergenic	ENSBTAG00000052185*	2
BTA13	20,203,933	6.2166e-07	Intergenic	ENSBTAG0000002675, ENSBTAG00000052951	0
BTA14	2,204,649	8.1819e-06	Intergenic	ENSBTAG00000048964*	1
	40,827,215	1.0856e-05	Intergenic	ENSBTAG00000039499	0
	79,535,150	3.2796e-06	Intergenic	None	0
BTA15	54,978,763	2.5073e-05	Intergenic	*MOGAT2*, ENSBTAG00000053794, *LOC785379*, ENSBTAG00000017443, ENSBTAG00000015091, ENSBTAG00000052936	1
	73,049,607	1.3516e-05	Intergenic	None	0
BTA17	4,778,666	1.7939e-05	Intron	*FHDC1*, ***ARFIP1***	1
	7,455,065	4.1422e-05	Intron	***LRBA***	0
	32,848,516	5.4123e-07	Intergenic	None	0
	36,481,658	9.3183e-06	Intron	***FSTL5***	1
BTA18	61,968,892	2.8497e-05	Intergenic	*EPN1*, *U2AF2*, *CCDC106*, *ZNF581*, *ZNF580*, *ZNF524*, *ZNF784*, *FIZ1*, *ZNF579*, *SBK2*, *SSC5D*, *NAT14*, *ZNF628*, *C19orf85*, ENSBTAG00000050011, *ISOC2*, *SHISA7*	0
	63,182,112	3.3624e-05	Intergenic	ENSBTAG00000049178, *RPS9*, *TSEN34*, *MBOAT7*, *TMC4*, *LENG1*, *CNOT3*, *PRPF31*, *TFPT*, *NDUFA3*	6
BTA19	59,744,349	4.8366e-06	Intergenic	None	0
BTA20	56,959,618	2.3978e-05	Intron	***MARCHF11***	4
	60,163,003	4.01e-05	Intergenic	None	0
BTA21	16,945,728	2.5982e-05	Intergenic	*AGBL1*	0
	60,917,828	2.6523e-06	Intron	***C21H14orf132***	0
BTA22	56,807,677	1.3049e-05	Intron	***PPARG***, ENSBTAG00000052393*	0
BTA23	2,330,898	2.7799e-05	Intergenic	None	0
	28,726,064	3.9504e-06	Upstream	*JSP.1*, ENSBTAG00000037421, ***BOLA***, *TRIM26*, *TRIM15*, *TRIM10*	0
BTA24	45,122,975	4.3551e-05	Intergenic	None	0
	57,019,331	7.1833e-06	Intergenic	*ATP8B1*	1
BTA25	8,675,209	3.4891e-05	Intron	***GRIN2A***	0
	8,702,138	2.0848e-06	Intergenic		
	27,413,783	6.8667e-06	Exon	*ITGAM*, ***ITGAX***, *ITGAD*, *COX6A2*, *ARMC5*, *TGFB1I1*	1
BTA27	24,754,025	1.5149e-05	Intron	*CLDN23*, ***LOC527981***	1
	43,998,949	1.466e-06	Intergenic	*ZNF385D*	1
BTA28	19,657,628	8.5e-06	Intron	*JMJD1C*, ***REEP3***	0
BTA29	13,752,854	2.5963e-05	Intergenic	None	0
	37,010,381	1.9927e-05	Intergenic	ENSBTAG00000050640*, *SNX19*, ENSBTAG00000022460, *MS4A8*, *MS4A18*	2

^a^Candidate genes are represented by gene symbol when available, otherwise by the Ensembl gene ID. The long noncoding RNA genes are marked by asterisk (*). The genes directly linked to SNPs are further denoted in bold

^b^The number of known and reproduction-associated QTL found in Cattle QTL Database v43 (www.animalgenome.org)

Eight GO terms and one KEGG pathway were significantly enriched for the 124 identified candidate genes (Fig. S3). Acylglycerol O-acyltransferase activity and diacylglycerol metabolism were the most significantly associated biological functions, which were linked to four genes (Monoacylglycerol O-acyltransferase 2, *MOGAT2*; 2-acylglycerol O-acyltransferase 2, *LOC785379*; ENSBTAG00000017443; ENSBTAG00000015091) within the region of BTA15:54.88–55.08 Mb and to one gene (Membrane bound O-acyltransferase domain containing 7, *MBOAT7*) within the region of BTA18:63.08–63.28 Mb. Three GO terms of “integrin complex”, “protein complex involved in cell adhesion”, and “plasma membrane signaling receptor complex” were linked to six genes on BTA25 (Glutamate ionotropic receptor NMDA type subunit 2A, *GRIN2A*; Integrin subunit alpha M, *ITGAM*; Integrin subunit alpha X, *ITGAX*; Integrin subunit alpha D, *ITGAD*), BTA18 (Shisa family member 7, *SHISA7*), and BTA10 (Integrin subunit alpha 11, *ITGA11*). The KEGG pathway of “Olfactory transduction” was also associated with 12 genes on BTA5 and BTA10.

### Multiple-trait analysis for sire traits

Eighty-four significant SNPs were associated with sire traits, which were broadly distributed across 24 chromosomes (Fig. 2 and Table 4). The three chromosomes that had the highest numbers of significant SNPs were BTA7, BTA17, and BTA22. The three most significant SNPs were located on BTA4, BTA5, and BTA26. There were one, 32, and one significant SNP located within the exonic, intronic, and upstream/downstream regions of 30 protein-encoding genes, respectively. Other 104 protein-encoding and 13 lncRNA genes were found within ± 100 Kb regions of 50 intergenic SNPs. Seventy-three QTL regions were determined from the 84 significant SNPs, and 31 of them (42.5%) overlapped with 72 previously reported QTL associated with reproduction traits in sires such as scrotal circumference, daughter pregnancy rate (DPR), CR, IPC, and interval to first estrus after calving (IFEC); Table S6. Among all the 147 candidate genes found for sire reproduction traits, only one GO term of “N-acetyl-beta-D-galactosaminidase activity” was shown to be significantly enriched (Fig. S3). Two genes located on BTA20, including *HEXB* (Hexosaminidase subunit beta) and *LOC786974* (Beta-hexosaminidase subunit beta) were also involved in this biological function.

**Table 4:** Significant SNPs and candidate genes from multiple-trait analysis for four sire fertility traits

Chr	Position (bp)	*P* values	Genomic location	Candidate genes (within ± 100 kb)^a^	N of QTL^b^
BTA3	147,935	2.2433e-05	Intergenic	ENSBTAG00000000584, *TBX19*	0
	71,522,570	2.1323e-05	Intergenic	—	0
BTA4	18,040,294	2.4781e-05	Intergenic	—	0
	47,200,188	2.5485e-05	Intergenic	*SYPL1*	0
	59,888,193	9.0367e-06	Intron	***ELMO1***	0
	82,878,864	9.7487e-07	Intergenic	*LSM8*	3
BTA5	6,106,039	2.7083e-06	Intergenic	*ZDHHC17*	0
	13,310,594	9.9233e-07	Intergenic	—	0
	32,058,789	1.0382e-05	Intergenic	*H1-7*, ENSBTAG00000032429, ENSBTAG00000032428, *CCDC184*, *ASB8*, *PFKM*	0
	53,516,007	2.3608e-05	Intergenic	—	6
BTA6	7,494,351	1.3907e-05	Intergenic	ENSBTAG00000051854*	2
	16,046,562	2.6665e-05	Intergenic	—	0
BTA7	56,985,760	2.23e-05	Intron	***PRELID2***, *GRXCR2*	0
	64,117,199	1.5768e-05	Intergenic	—	2
	65,880,766	2.3775e-05	Intergenic	ENSBTAG00000053039*, LARP1	0
	65,887,207	2.5969e-06	Intergenic		
	68,359,300	1.6201e-05	Intergenic	ENSBTAG00000030297, *TIMD4*, ENSBTAG00000050582*, ENSBTAG00000049542*	0
	68,430,783	1.5884e-05	Intergenic		
	69,170,746	2.7112e-05	Intron	*CYFIP2*, *NIPAL4*, ***ADAM19***, *SOX30*	0
	83,320,343	2.8225e-06	Intron	***XRCC4***, *VCAN*	1
	100,056,219	8.7549e-06	Intron	***ST8SIA4***	2
BTA9	15,828,286	7.3866e-06	Intergenic	—	2
	15,843,031	2.5491e-05	Intergenic		
	74,655,065	1.7472e-05	Intron	***MAP3K5***	4
BTA10	13,081,096	2.3448e-05	Intron	***MEGF11***	0
	55,196,388	9.9519e-06	Intergenic	*RSL24D1*	0
	77,838,554	2.8177e-05	Downstream	***FUT8***	0
	77,968,781	1.3016e-05	Intergenic		
BTA11	58,988,314	1.1679e-05	Intergenic	—	0
	63,929,970	1.3355e-05	Intergenic	—	0
BTA12	60,487,742	4.3234e-06	Intergenic	—	0
	63,535,854	1.6981e-05	Intergenic	—	0
	63,538,393	2.1305e-05	Intergenic		
BTA13	13,568,203	2.023e-05	Intergenic	—	0
	14,812,273	1.4407e-05	Intergenic	—	0
	41,317,365	1.2933e-05	Intergenic	—	0
	53,595,922	1.5122e-05	Intron	ENSBTAG00000054594, ENSBTAG00000011638, ENSBTAG00000054447, ENSBTAG00000027221, ***GINS1***, *PCMTD2*, *MYT1*	2
BTA14	78,863,167	3.3468e-05	Intergenic	—	0
BTA15	30,636,699	1.2166e-05	Intergenic	*TRIM29*	3
	60,970,873	2.5168e-05	Intergenic	*FSHB*, *ARL14EP*	2
BTA16	23,387,972	1.0853e-05	Intergenic	*SLC30A10*, *EPRS1*	0
BTA17	18,332,441	2.8154e-05	Intron	*RAB33B*, ***NAA15***, *NDUFC1*, ***MGARP***, ENSBTAG00000015811, *ELF2*	3
	18,371,778	2.706e-05	Intron		
	18,408,888	2.8364e-05	Intron		
	18,458,616	2.3968e-05	Intergenic		
	33,971,794	2.9933e-05	Intergenic	ENSBTAG00000052376*	5
	48,501,570	3.3186e-05	Intron	***TMEM132C***	2
	73,040,719	1.7853e-05	Intergenic	*TANGO2*, ENSBTAG00000049742*, *DGCR8*, *TRMT2A*, ENSBTAG00000049062, *RANBP1*, *ZDHHC8*, *CCDC188*, ENSBTAG00000052630*, *RTN4R*, *PRODH*, *DGCR6L*	8
BTA19	3,507,732	6.9599e-06	Intergenic	—	0
	6,425,261	2.0337e-05	Intron	*PCTP*, ENSBTAG00000053407, **ENSBTAG00000039563**	0
	23,803,632	4.6828e-05	Intron	***RAP1GAP2***, ENSBTAG00000049401, *OR1D3B*, *OR1D3*	2
	23,811,867	3.2146e-05	Intron		
BTA20	6,792,445	2.1451e-05	Intron	*FAM169A*, *NSA2*, ***GFM2***, *HEXB*, *LOC786974*	0
	44,069,982	8.8349e-06	Intergenic	—	0
	53,664,202	1.0238e-05	Intron	***CDH18***	0
BTA21	3,550,200	1.2359e-05	Intergenic	—	0
BTA22	529,021	3.8026e-05	Intron	**VOPP1**, **LANCL2**	0
	756,892	2.5524e-05	Intron		
	37,660,216	4.9522e-05	Intergenic	*THOC7*, *C22H3orf49*, ENSBTAG00000051113*, *SNTN*	3
	42,471,770	2.3695e-05	Intron	***CFAP20DC***	3
	44,200,445	1.8137e-06	Intron	***ARHGEF3***	0
	59,781,347	1.759e-05	Intron	***MGLL***, *ABTB1*, *PODXL2*	0
BTA24	1,516,807	1.8306e-05	Intergenic	—	0
	20,595,336	4.1116e-05	Intron	*FHOD3*	1
	57,446,121	5.5515e-06	Intron	***NEDD4L***, ENSBTAG00000051924*	0
	57,476,264	2.8706e-05	Exon		
	61,476,879	2.1094e-05	Intron	***BCL2***	1
BTA25	26,121,115	1.2696e-05	Intron	*EIF3CL*, *CLN3*, ENSBTAG00000050361, *IL27*, *NUPR1*, *SGF29*, ***SULT1A1***, *SLX1A*, ENSBTAG00000008632, *CORO1A*, *MAPK3*, *GDPD3*, *YPEL3*, *TBX6*, *PPP4C*, *ALDOA*, ENSBTAG00000050743	1
	27,554,453	2.3765e-05	Intergenic	*ITGAD*, *COX6A2*, *ARMC5*, *TGFB1I1*, *SLC5A2*, *RUSF1*, *AHSP*, *OR7A53*, *OR7A153*, *SEPTIN14*, *ZNF713*, *MRPS17*	1
	39,147,647	6.6288e-05	Intron	*MMD2*, ENSBTAG00000051446, ***RADIL***, *PAPOLB*, *AP5Z1*, ENSBTAG00000050145*, *FOXK1*	1
	40,290,599	1.0271e-05	Intergenic	*CARD11*	0
	40,356,262	5.4215e-05	Intergenic		
BTA26	10,927,671	1.4324e-06	Intergenic	ENSBTAG00000054811*, *LIPA*	1
	27,142,712	2.691e-05	Intergenic	ENSBTAG00000055185*	0
	30,565,345	4.8874e-05	Intergenic	*XPNPEP1*, ENSBTAG00000054944, *ADD3*	0
	48,212,324	5.1946e-05	Intergenic	ENSBTAG00000053738*	5
BTA27	13,351,474	3.8108e-05	Intron	**ENSBTAG00000047749**	1
	25,506,261	6.2859e-05	Intron	***TNKS***	1
BTA28	5,066,852	2.9294e-05	Intergenic	—	1
	19,499,175	7.0726e-06	Intron	***JMJD1C***	0
	29,298,316	2.2431e-06	Intron	*NUDT13*, *ECD*, *FAM149B1*, *DNAJC9*, ***MRPS16***, *CFAP70*, *ANXA7*	0
	37,896,374	1.0502e-05	Intron	***NRG3***	1
BTA29	4,807,970	5.4737e-05	Intergenic	—	1
	14,612,904	3.5086e-05	Intergenic	—	1

^a^Candidate genes are represented by gene symbol when available, otherwise by the Ensembl gene ID. The long noncoding RNA genes are marked by asterisk (*). The genes directly linked to SNPs are further denoted in bold

^b^The number of known and reproduction-associated QTL found in Cattle QTL Database v43 (www.animalgenome.org)

### Multiple-trait analysis for the combined trait categories

When the 14 female fertility and reproduction traits were combined (heifers and cows), 96 significant SNPs were identified across 28 chromosomes (Fig. 2 and Table S7). The three most significant SNPs were located on BTA23, BTA22, and BTA4, respectively. Ninety-six QTL were detected and 25 of them overlapped with 60 previously reported reproduction-associated QTL in cattle (Table S8). There were 171 candidate genes found, in which, nine GO terms and one KEGG, such as Phagosome (KEGG:04,145) and rough endoplasmic reticulum (GO:0,005,791), were significantly enriched for them (Table S9).

The multiple-trait chi-square statistics was also applied to all 18 traits of heifers, cows, and sires combined. Eighty-six significant SNPs and 178 candidate genes were identified across 26 chromosomes (Fig. 2 and Table S10). All significant SNPs were clustered into 77 QTL, and 27 of them (35%) overlapped with previously reported reproduction-associated QTL in cattle for traits, such as CR, calving ease (CE), DPR, and IPC (Table S11). The functional enrichment analyses revealed four GO terms, including “2-acylglycerol O-acyltransferase activity”, “Diacylglycerol biosynthetic process”, “Acylglycerol O-acyltransferase activity”, and “O-acyltransferase activity” (Table S9). For these biologically relevant genes identified for heifer, cow, and sire traits in this study, we also performed a protein–protein interaction analysis, but no direct functional interactions among them were observed as shown in Fig. S4.

## Discussion

### Refined studies on fertility and reproduction traits in dairy cattle

Due to the great economic importance of fertility and reproduction traits in cattle industry, GWAS have been frequently carried out especially since genotyping become affordable for large sample sizes. Besides the fact that SNP panels have been used in former GWAS of cattle fertility and reproduction traits [27–39], whole-genome sequence variants recently began to be used to refine QTL boundaries and identify causal genes [18–20]. Therefore, the imputed or real WGS variants is being increasingly used for GWAS, especially for traits with low heritability and that are highly polygenic. Because of intrinsic correlations among various indicator traits of fertility and reproduction, it is preferable to perform multiple-trait GWAS to detect pleiotropic variants and increase the detection power of important variants. The classical methods of multiple-trait GWAS [40, 41] are difficult to implement in practice, especially on large-scale datasets due to computing requirements. Alternatively, an approximate method was proposed by Bolormaa et al. [24], which efficiently computes a multiple-trait chi-square statistic from estimated SNP effects from single-trait GWAS. In this study, we used imputed WGS variants and this approximate method for multiple-trait GWAS of fertility and reproduction traits in Holstein cattle, aiming to refine the associated pleiotropic variants, candidate genes, and QTLs.

### Polygenic nature of fertility and reproduction traits

Most fertility and reproduction traits are lowly heritable in cattle. Berry et al. [21] reported a summary of heritability estimates below 0.05 for most female traits and 0.05–0.22 for most male traits. Similarly, the reported QTL and candidate genes in literature have been found to be extensively distributed across genome, and few of them could be repeated across studies [3, 12]. In this study, a similar genetic landscape was observed, as all autosomes harbored significant SNPs for one or more traits analyzed, being consistent with the polygenic nature of these traits. Numerous fertility and reproduction indicator traits have been defined for measuring reproductive performance at different reproductive stages in female cattle [3, 21]. However, some of these traits are by definition related with each other, such as CI is greatly determined by the interval from calving to conception. Therefore, pleiotropic variants are expected for these traits [10, 42]. Overall, we found that the number of significant SNPs from multiple-trait analysis was less than the cumulative sum of separate single-trait analysis; and similar results were found in previous GWAS studies on fertility and reproduction [e.g., 27].

### Differences between heifer and cow traits

It is well known that the genetic basis and physiological processes underlying reproduction differ between heifers and lactating cows. In general, heifer traits have higher heritability, but lower genetic correlations with each other compared to cow traits [43, 44]. There are also only moderate genetic correlations between heifer and cow traits, which are lower than between cow traits in different parities [23, 44–46]. Likewise, both ovarian structures and circulating steroids (such as serum concentrations of progesterone and estradiol) were observed to act differently between heifers and cows. In addition, lactating cows have lower circulating steroid concentrations, but larger ovulatory follicles and luteal structures than heifers [47, 48].

In previous GWAS for fertility and reproduction traits, different variants, candidate genes, and QTL were found significantly associated with heifers and cows [20, 26, 27, 37]. For instance, Fang et al. [26] found a significant QTL on BTA17 associated with interval from first to last insemination (IFLI) and non-return rate (NRR) in heifers, but not in cows. For NRR, distinct significant QTL regions on BTA17 were also suggested for heifers and cows [20]. Liu et al. [27] analyzed four fertility traits (IFLI, FSC, IPC, and NRR) measured on both heifers and cows, and found that none of the significant QTL overlapped and most of these QTL were located on distinct chromosomes. In this study, our multiple-trait analysis found that there were only two candidate QTL (around at BTA9:21.35 Mb and BTA23:28.63 Mb) overlapping between heifers and cows. Furthermore, our functional analyses of candidate genes also suggested differences between heifer and cow traits. The candidate genes found for heifers were significantly involved in immunity-associated biological functions, whereas candidate genes found for cows were associated with acylglycerol O-acyltransferase activity and diacylglycerol metabolism.

### QTL and candidate genes found for heifer traits

The mean CR of Holstein heifers was estimated to be 56.3% in the United States, which is influenced by the age at breeding, month of breeding, age of service sire, and other factors [45]. Accordingly, our multiple-trait analysis for heifer traits suggested dozens of associated QTL that are broadly distributed across three quarters of all included chromosomes. Notably, one-third of these QTL overlapped with previously identified reproduction-related QTL in cattle. For example, the QTL located on BTA3:89.78–89.98 Mb was supported by a QTL previously reported to be associated with the commencement of LA and proportion of cows in LA between 25 and 60 days in milk [19], and with CR [49]. There were five QTL previously reported to be associated with DPR, CR, and IFEC [50–52], overlapping with one QTL on BTA9:95.88–96.08 Mb found in this study. The QTL located on BTA12:77.58–77.78 Mb overlapped with four previously reported QTL associated with CE, DPR, and stillbirth [31, 53]. Furthermore, we identified many novel QTL in this study, such as BTA13:41.92–42.15 Mb, BTA18:39.57–40.01 Mb, BTA19:53.68–53.88 Mb, and BTA22:11.29–11.84 Mb, which showed strong association with heifer traits.

For the CR at first service and the number of times bred in Holstein heifers, Galliou et al. [54] found that three out of the five most significant pathways were involved in immune system regulation, which was consistent with our results. More importantly, the non-classical MHC I gene of *BoLA-NC1*, found in this study and involved in the KEGG pathway of allograft rejection, was suggested to increase maternal immunity against the fetus [55]. Another non-classical MHC I gene (*JSP.1*) was found to play a crucial role during early pregnancy in heifers in a previous study [56]. Similarly, Melo et al. [57] found that the MHC class II genes were significantly associated with pregnancy success in Nellore cows. As the link between autophagy and reproduction has been acknowledged [58], another autophagy-related gene of phosphatidylinositol 3-kinase catalytic subunit type 3 (*PIK3C3*) was identified in this study. These findings suggest the importance of immunological tolerance of dam to fetus and other immune responses in establishing successful pregnancy.

Notably, a QTL, that was located on BTA9:95.88–96.08 Mb and supported by five previously reported QTL, contained four candidate genes having biological implications in reproduction, including the superoxide dismutase 2 (*SOD2*) for meiotic defects in mouse oocytes [59], WT1 associated protein (*WTAP*) for spermatogenesis in mice [60], acetyl-CoA acetyltransferase 2 (*ACAT2*) for DPR in Holstein [52], and t-complex 1 (*TCP1*) for mediating sperm-oocyte interaction in mice [61]. On BTA22, there were five suggestive candidate genes, including the transforming growth factor beta receptor 2 (*TGFBR2*) for female fertility in pigs and mice [62, 63], DLEC1 cilia and flagella associated protein (*DLEC1*) and solute carrier family 22 member 14 (*SLC22A14*) regulating male fertility in mice [64, 65], activin A receptor type 2B (*ACVR2B*) associated with premature ovarian failure in human [66], and 6-phosphofructo-2-kinase fructose-2,6-biphosphatase 4 (*PFKFB4*) involved in spermatogenesis in mice [67]. Two genes, the tripartite motif containing 27 (*TRIM27*) and *PRP14* on BTA23, were suggested to be associated with male and female fertility, respectively [68, 69]. Other positional candidate genes included the transmembrane protein with EGF like and two follistatin like domains 2 (*TMEFF2*) on BTA2 involved in early oocyte development in human [70] and associated with bull sperm morphometry [71], HECT, C2 and WW domain containing E3 ubiquitin protein ligase 1 (*HECW1*) on BTA4 required for estrogen-induced degradation of Scribble [72], cystatin 8 (*CST8*) on BTA13 involved in sperm maturation [73], dynein axonemal heavy chain 17 (*DNAH17*) on BTA19 associated with human sperm fertility [74, 75], ADAM metallopeptidase domain 3A (*ADAM3A*) on BTA27 regulating sperm migration in mice [76], and zona pellucida glycoprotein 4 (*ZP4*) on BTA28 regulating embryo development in rabbits [77]. Taken together of the experimental evidence in literature, these genes have been supported to hold biological roles in reproduction of humans and other species.

### QTL and candidate genes found for cow traits

Our multiple-trait analysis for cow traits revealed that all significant QTL were broadly distributed across more than 85% of the chromosomes and one-third of them were supported by previously reported QTL in cattle. For instance, one QTL located on BTA18:63.08–63.28 Mb was previously reported significantly associated with CI and stillbirth [36, 78]; one QTL located on BTA20:56.86–57.06 Mb was supported by two previously reported associations with CR, FSC, and IPC [49, 54]; and one QTL located on BTA5:61.82–62.02 associated with FSC and CR [79]. In addition, many novel QTL identified in this study harbored candidate genes that have biological functions impacting reproduction according to the literature.

Our analyses of candidate genes for cow traits revealed functional implications that are in contrast to those for heifers. Instead of immune system, the most significant functions were associated with fatty acid metabolism, such as the acylglycerol O-acyltransferase activity and diacylglycerol metabolism. Mattos et al. [80] comprehensively reviewed effects of dietary fatty acids on reproduction in ruminants, which include the influences on ovarian follicle and corpus luteum function via improved energy status, and the synthesis of reproductive hormones, such as steroids and prostaglandins. In addition, the maternal lipid metabolism during pregnancy positively influence fetal growth [81, 82]. Many genes involved in fatty acid metabolism were similarly found to be associated with reproductive performance in Nellore cattle [57]. The seasonal changes in Holstein fertility was related to fatty acid composition of follicles [83]. Furthermore, the relationship between dietary fatty acids and ovarian function was experimentally found in Holstein cows [84].

Interestingly, numerous candidate genes identified in this study also have the known biological functions associated with reproduction in literature. Two genes (*GRIN2A* and *ITGAX*) located on BTA25 were associated with CTFS in Iranian Holstein cattle [85] and with altered expression in polycystic ovary syndrome in women [86], respectively. There were four candidate genes located on BTA8, including the kinesin family member 13B (*KIF13B*) involved in oocyte meiosis [87], annexin A1 (*ANXA1*) with regulatory functions during early pregnancy in mice [88] and differentially expressed between less fertile and normally fertile Holstein [89], proprotein convertase subtilisin/kexin type 5 (*PCSK5*) that contributed to ovarian follicle development in rats [90], and zinc finger protein 483 (*ZNF483*), which is associated with age of puberty in Brahman heifers [91]. Two genes, the fem-1 homolog B (*FEM1B*) and sperm equatorial segment protein 1 (*SPESP1*) on BTA10, were reported to be involved in polycystic ovary syndrome in humans [92] and required for fully fertile sperm in mice [93], respectively. Four genes were associated with different reproduction traits, including cell adhesion molecule 2 (*CADM2*) on BTA1 with the number of piglets born dead in pigs [94], calcium voltage-gated channel auxiliary subunit beta 4 (*CACNB4*) on BTA2 with CTFS in Iranian Holstein Cattle [85], ST6 N-acetylgalactosaminide alpha-2,6-sialyltransferase 5 (*ST6GALNAC5*) on BTA3 with fertility index in Holstein cattle [95], and ATP/GTP binding protein like 1 (*AGBL1*) on BTA21 with out of season lambing in sheep [96]. Other suggestive candidate genes included the protein tyrosine phosphatase receptor type Z1 (*PTPRZ1*) on BTA4 differentially expressed between high- and low-fertility Holstein cows [97], LPS responsive beige-like anchor protein (*LRBA*) on BTA17 differentially methylated between high- and low-fertility bulls [98], peroxisome proliferator activated receptor gamma (*PPARG*) on BTA22 involved in the release of oocytes each estrous cycle [99], and jumonji domain containing 1C (*JMJD1C*) on BTA28 required for long-term maintenance of male germ cells in mice [100].

### QTL and candidate genes found for sire traits

In dairy cattle, the genetic evaluation of male reproduction has received much less attention in comparison with female traits [101, 102]. Sire CR in Holstein was included in two previous GWAS studies [103, 104], which revealed significant SNPs located on multiple chromosomes. In this study, we included four sire traits and found that the number and genomic distribution of QTL are comparable with or higher than that in females. Interestingly, more than 40% of these QTL identified in this study overlapped with previously reported reproduction-related QTL in cattle. For instance, all four QTL located on BTA17 (BTA17:18.23–18.56 Mb, BTA17:33.87–34.07 Mb, BTA17:48.40–48.60 Mb, and BTA17:72.94–73.14 Mb) overlapped with previous reports for DPR, IPC, CE, stillbirth, IFEC, and NRR [31, 34, 49, 50, 54] and three out of four QTL on BTA25 (BTA25:26.02–26.22 Mb, BTA25:27.45–27.65 Mb, and BTA25:39.05–39.25 Mb) overlapped with previous reports for age at first calving (AFC) and CR [105, 106].

Only one GO term (N-acetyl-beta-D-galactosaminidase activity) was significantly enriched among all candidate genes of sire traits. The two involved genes (*HEXB* and *LOC786974*) on BTA20 have no functional evidence in literature with respective to reproduction. However, we found many novel candidate genes that have direct functional implications in reproduction. For example, the engulfment and cell motility 1 (*ELMO1*) gene on BTA4 was found to play crucial roles in clearance of apoptotic germ cells and spermatogenesis in mice [107]. Two genes, *JMJD1C* and cilia and flagella associated protein 70 (*CFAP70*) on BTA28, are required for long-term maintenance of germ cells in mice [100] and are associated with multiple morphological abnormalities of sperm flagella in human [108]. Notably, both *ELMO1* and *JMJD1C* genes were suggested by the significant SNPs located in intronic regions. One well-known candidate gene on BTA15 (follicle stimulating hormone subunit beta, *FSHB*), associated with semen quality and fertility in bulls [109], was also identified in this study.

There were four suggestive candidate genes on BTA7, including the PRELI domain containing 2 (*PRELID2*) associated with fertility traits in pigs [110], ADAM metallopeptidase domain 19 (*ADAM19*) differentially expressed between different quality of bovine blastocysts [111], SRY-box transcription factor 30 (*SOX30*) required for male fertility in mice [112, 113], and versican (*VCAN*) involved in embryo implantation in rabbits [114]. Three candidate genes were found on BTA17, including *RAB33B*, member RAS oncogene family (*RAB33B*), associated with freezability in boar spermatozoa [115], transport and golgi organization 2 homolog (*TANGO2*) influencing bull fertility [116], and DGCR8 microprocessor complex subunit (*DGCR8*) involved in human spermatogenesis [117]. On BTA25, the mitogen-activated protein kinase 3 (*MAPK3*), septin 14 (*SEPTIN14*), and poly(A) polymerase beta (*PAPOLB*) were found to regulate ovulation in mice [118], spermatogenesis in humans [119], and spermatogenesis in mice [120], respectively. Other suggestive candidate genes included the synaptophysin like 1 (*SYPL1*) on BTA4 contributing to sperm maturation in sheep [121], multiple EGF like domains 11 (*MEGF11*) on BTA10 associated with lifetime productivity in pigs [122], fucosyltransferase 8 (*FUT8*) on BTA10 differentially expressed between matured oocytes from older and younger women [123], myelin transcription factor 1 (*MYT1*) on BTA13 involved in reduced fertility of anovular dairy cows [124], Rho guanine nucleotide exchange factor 3 (*ARHGEF3*) on BTA22 associated with number of piglets weaned [125], monoglyceride lipase (*MGLL*) on BTA22 influencing metabolism of endocannabinoids in bovine endometrium [126], NEDD4 like E3 ubiquitin protein ligase (*NEDD4L*) on BTA24 associated with AFC in buffaloes [127], and BCL2 apoptosis regulator (*BCL2*) on BTA24 influencing oocyte and early embryo survival in humans [128].

### Combining multiple trait categories

We combined multiple trait categories for multiple-trait analysis for exploiting potential pleiotropic effects for fertility and reproduction traits. When the 14 female traits were analyzed together, 10 out of the 33 suggestive candidate genes in heifer and cow traits were identified. In addition, 15 out of the 54 suggestive candidate genes in heifers, cows, and sires were identified when also combining the four male traits together. There were five additional identified candidate genes in the combined analysis that have biological implications in reproduction in the literature, including Cbp/p300 interacting transactivator with Glu/Asp rich carboxy-terminal domain 2 (*CITED2*) on BTA9, cadherin 1 (*CDH1*) on BTA18, fanconi anemia complementation group M (*FANCM*) on BTA23, DPY30 domain containing 1 (*DYDC1*) on BTA28, and neural EGFL like 2 (*NELL2*) on BTA5 [129–133]. Among them, *NELL2* was recently found to be involved in lumicrine system essential for testis-epididymis-spermatozoa signaling and male fertility [133]. Furthermore, three chromosomes (BTA3, BTA11, and BTA24) no longer contained significant regions when all 18 traits were analyzed together. A possible explanation for these findings is that the increased amount of information might have removed spurious associations.

### Implications of the study

The results of this study have three main implications. First, we enhanced compelling evidence that traits measured in heifers, cows, and sires hold relatively distinct polygenic nature, therefore having specific evaluation is needed in breeding programs. Second, the QTL and candidate genes found in this study can be specifically incorporated into genomic prediction models by giving greater weights to the more important markers. Furthermore, SNPs located in the relevant genomic regions identified can also be included in commercial genotyping platforms to increase the accuracy of genomic prediction. Third, we suggested several novel candidate genes associated with fertility and reproduction traits in cattle, which should be further investigated in sequel studies. Additionally, the use of closer to biology phenotypes that better capture the biological mechanisms underlying the fertility and reproduction traits and the use of multi-omic data (e.g., transcriptomics, metabolomics, epigenomics) can further facilitate the mapping of QTL and variants associated with fertility and reproduction. Future studies could further investigate the most promising candidate genes using gene editing or gene knock-out experiments and real WGS data (instead of imputed WGS), which would enable the investigation of rare alleles (usually removed due to low imputation accuracy) and structural variation in the genome (e.g., copy number of variants, insertions, deletions). For the significant intergenic SNPs found in this study, their possible functional roles will be explored when a comprehensive annotation of genomic regulatory elements are available.

## Conclusions

The multiple-trait GWAS of 18 fertility and reproduction traits in North American Holstein cattle revealed several QTL and candidate genes associated with heifer, cow, and sire traits. These QTL were broadly distributed across the entire genome, which are consistent with the polygenic nature of fertility and reproduction traits. The biological functions of immune response and fatty acid metabolism were significantly enriched in heifer and cow traits, respectively, whereas no known functional enrichment was found for sire traits. The most important chromosomes, which had three or more significant QTL, were BTA22 and BTA23 for heifer traits, BTA8 and BTA17 for cow traits, and BTA4, BTA7, BTA17, BTA22, BTA25, and BTA28 for sire traits. Several candidate genes that have not been previously reported in cattle and other livestock were strongly suggested for heifer (*SOD2*, *WTAP*, *DLEC1*, *PFKFB4*, *TRIM27*, *HECW1*, *DNAH17*, and *ADAM3A*), cow (*ANXA1*, *PCSK5*, *SPESP1*, and *JMJD1C*), and sire (*ELMO1*, *CFAP70*, *SOX30*, *DGCR8*, *SEPTIN14*, *PAPOLB*, *JMJD1C*, and *NELL2*) traits. More than one-third of the QTL identified in this study overlapped with previously reported reproduction-related QTL in cattle, and more than 50 candidate genes were supported by functional implications in reproduction, as found in the literature. In addition, many important candidate genes were identified via significant SNPs located in their intronic regions. These observations indicate high detection power and mapping resolution of this study. Therefore, a comprehensive investigation on underlying genetic basis of fertility and reproduction in heifers, cows, and sires are provided in this study, whose findings can be used for improving genomic evaluation for fertility and reproduction traits in Holstein cattle.

## Methods

### Animals, phenotypes and genotypes

The phenotypic and genotypic datasets used in this study were provided by the Canadian Dairy Network (CDN), a member of Lactanet Canada (Guelph, ON, Canada). A total of 18 fertility and reproduction traits were analyzed in this study (Table 1). For all traits, dEBV were used as pseudo-phenotypes, which were computed following VanRaden et al. [134] and only dEBV with accuracy greater than 0.50 were kept for further analyses. The details of trait definitions and the effects included in the statistical model used to predict the original estimated breeding values (EBV) for each trait are reported in Oliveira Junior et al. [9].

Imputed WGS datasets containing 29,548,077 SNPs were available for 9,131 animals. The detailed genotype imputation process using the FImpute software [135], including the number of animals per SNP panel, methods, and QC, was described by Chen et al. [136]. In brief, genotype imputation was performed in two steps: (1) imputation from medium density panels (9,131 cows and 56,955 or 60,914 SNP; Illumina, San Diego, CA, USA) to the BovineHD panel [HD, 311,725 SNP after a preliminary quality control; Illumina, San Diego, CA, USA]; and (2) imputation from HD to WGS. The reference population for step 1 had 2,397 animals (from the same herds and Holstein population), whereas for step 2, there were 1,147 animals with WGS data (from the 1,000 Bull Genomes Project, which also included North American Holstein animals) [136]. An additional QC step was applied after imputation, which required the individual and genotype missing rates to be lower than 0.1 (this step was done as some markers were still missing after the imputation process), MAF to be higher than 0.01, and no extreme deviation from Hardy–Weinberg equilibrium (only retained SNPs with *P* > 1.0 × 10^–8^). The SNPs that were poorly imputed were also removed from the analyses. This was done in a previous step where accuracy of imputation was calculated based on genotype concordance rate and allelic R^2^ on a per-SNP basis, where the SNPs retained had a concordance rate and allelic R^2^ greater than 0.95 and 0.85, respectively. All QC were conducted using the PLINK software [137], after which an average of 5,576,878 SNPs were retained for single-trait GWAS using 3,803 to 5,986 animals (depending on the trait, as showed in Table 1).

### Single-trait association analyses

As all known fixed effects were fitted when predicting the EBVs used to compute the dEBVs, only SNP and polygenic effects were included in the mixed linear model used for the association analyses, i.e.:

$$\mathbf{y}=1\upmu +\mathbf{X}\mathrm{b}+\mathbf{Z}\mathbf{u}+\mathbf{e},$$

where $\mathbf{y}$ is the vector of dEBV for each analyzed trait; $1$ is a vector of ones; $\upmu$ is the overall mean; $\mathrm{b}$ is the fixed effect of the SNP tested for association, **X** is a vector containing the genotype score for the tested SNP; $\mathbf{u}$ is the random vector of polygenic effect with $\mathbf{u} \sim N(0, \mathbf{G}{\sigma }_{u}^{2})$, where $\mathbf{G}$ is the genomic-based relationship matrix (GRM), ${\sigma }_{u}^{2}$ is the additive genomic variance of polygenic effects; $\mathbf{Z}$ is the incidence matrix for $\mathbf{u}$; and $\mathbf{e}$ is a random vector of residual effects with $\mathbf{e} \sim N(0, \mathbf{I}{\sigma }_{e}^{2})$, where $\mathbf{I}$ is an identity matrix and ${\sigma }_{e}^{2}$ is the residual variance. The genetic relationship between individuals $j$ and $k$ in the GRM was computed as [138]:

$${G}_{jk}=\frac{1}{N}\sum_{i=1}^{N}\frac{({x}_{ij}-2{p}_{i})({x}_{ik}-2{p}_{i})}{2{p}_{i}(1-{p}_{i})},$$

where ${p}_{i}$ is the frequency of the reference allele for the *i*^th^ SNP; ${x}_{ij}$ and ${x}_{ik}$ are the numbers of copies of the reference allele for individuals $j$ and $k$, respectively; and $N$ is the total number of SNPs used. Instead of using all included SNPs, a total of 29 GRMs were constructed by randomly sampling 50,000 SNPs from the remaining 28 chromosomes (i.e., iterative process including all chromosomes except the one in which the analyzed SNP was located). These analyses were implemented using the GCTA software [138].

The strongly-linked SNPs were clumped out if they had high LD (*r*^2^ > 0.9) with another SNP that have a lower *P* in the GWAS, using the PLINK software [137]. This clumping process avoids overestimating the number of significant SNPs and was suggested to be preferable compared to the pruning method without considering the *P* values from GWAS [139]. To check if there was potential stratification due to the population structure, the genomic inflation factor (λ) [140] was evaluated, along with its 95% confidence interval.

### Multiple-trait chi-square statistics

Following Bolormaa et al. [24], the multiple-trait analysis was performed using five different trait categories (i.e., heifers, cows, sires, heifers and cows, and all traits), as shown in Table 1. The CA trait was simultaneously included into both heifer and cow categories as it is a sub-index incorporating other traits. For each category of $n$ traits, the multiple-trait chi-square statistics of a SNP was obtained as follows [24]:

$${\upchi }^{2}={{\varvec{t}}}_{i}^{^{\prime}}{{\varvec{V}}}^{-1}{{\varvec{t}}}_{i}$$

where ${{\varvec{t}}}_{i}$ is a $n\times 1$ vector of signed t-values for ${i}^{th}$ SNP (that is equal to the allele effect divided by its standard error) across the $n$ analyzed traits; ${{\varvec{t}}}_{i}^{^{\prime}}$ is the transpose of vector ${{\varvec{t}}}_{i}$ ($1\times n$); and ${{\varvec{V}}}^{-1}$ is the inverse of $n\times n$ correlation matrix, in which the pairwise correlations of traits were calculated over the estimated SNP effects. The null hypothesis that the SNP has no significant effect on any of the tested traits was tested based on the ${\upchi }^{2}$ distribution with $n$ degrees of freedom.

### Adjustment for multiple-hypotheses testing

Due to the polygenic architecture of fertility and reproduction traits, a large number of QTL are expect, therefore a Bonferroni correction at 5% chromosome-wise significance level [141] was carried, by dividing 0.05 by the number of independent chromosome segments (${M}_{e}$) to account for dependency among tests. The ${M}_{e}$ was calculated as follow [142]:

$${M}_{e}=\frac{2{N}_{e}L}{\mathrm{ln}\left({N}_{e}L\right)},$$

where ${N}_{e}$ is the effective population size, which was set to 66 according to a recent report in North American Holstein [143]; $L$ is chromosome length expressed in centi-Morgans (cM; one cM was considered to be equivalent to 1 Mb). Thus, SNPs were considered as statistically significant if their ${-\mathrm{log}}_{10}(P)$ was higher than the chromosome-wide threshold [$-{\mathrm{log}}_{10}(0.05/{M}_{e})$], which ranged from 4.15 to 4.65 depending on the chromosome (average ± SD = 4.39 ± 0.12).

### QTL, positional candidate genes, and functional analyses

The QTL were defined as chromosomal regions of ± 100 Kb around the significant SNPs, therefore, considering a flanking distance where, on average, high LD is expected in North American Holsteins [144]. The identified QTL were compared to previously reported QTL in the Cattle QTL Database – Release 43 [145]. To most effectively avoid missing the potential causal genes, all positional candidate genes within QTL region, including protein-encoding and lncRNA, were retrieved using the biomaRt R package [146]. The ARS-UCD1.2 assembly (https://ensembl.org/Bos_taurus/Info/Index) was used as the reference genome. Functional enrichment analyses of the candidate genes were conducted using the g:GOSt function of the g:Profiler web server [147], including the target datasets of the GO terms [148] and KEGG pathways [149]. The default parameters and methods for adjusting for multiple hypotheses testing were used, targeting an adjusted 5% level of significance. For the biologically suggested candidate genes, we also performed a protein–protein interaction analysis using the STRING software [150].

## Supplementary Information

**Additional file 1: TableS1.** Position distribution and minor allele frequencies of SNPs that passed thegenotype quality control; **Table S2.** Distribution of significant SNPs of single-traitGWAS; **Table S3.** Numbers of quantitative trait loci revealed by single-traitGWAS and their overlapping among traits; **Table S4.** The previously reported andreproduction-associated QTL for the significant SNPs revealed by multiple-traitanalysis of six heifer traits; **Table S5.** The previously reported andreproduction-associated QTL for the significant SNPs revealed by multiple-traitanalysis of nine cow traits; **Table S6.** The previously reported andreproduction-associated QTL for the significant SNPs revealed by multiple-traitanalysis of four sire traits; **Table S7.** Significant SNPs and candidate genesfrom multiple-trait analysis of 14 heifers and cows’ traits; **Table S8.** Thepreviously reported and reproduction-associated QTL for the significant SNPsrevealed by multiple-trait analysis of 14 heifer and cow traits; **Table S9.** Thesignificantly enriched GO terms and KEGG from multiple-trait analysis of 18heifers, cows, sire traits; **Table S10.** Significant SNPs and candidate genesfrom multiple-trait analysis of 18 heifers, cows, sire traits; **Table S11.** Thepreviously reported and reproduction-associated QTL for the significant SNPsrevealed by multiple-trait analysis of 18 heifer, cow, and sire traits; **Figure S1.** Manhattan plots (left) and Quantile-quantile plots (right) of thesingle-trait GWAS results based on imputed WGS data; **Figure S2.** Numbers of significant SNPs foreach trait category and their overlaps found by multiple-trait analysis. **Figure S3.** Significantly enrichedbiological functions of candidate genes revealed by multiple-trait analysis. **Figure S4.** Potential protein-protein interaction among biologically relevantgenes that were identified for heifer, cow, and sire traits in this study.

## Data Availability

All the data supporting the results of this study are included in the article and in the Additional file.

## Abbreviations

AFC: Age at First Calving
BTA: Bos Taurus Autosomes
CE: Calving Ease
CI: Calving Interval
CR: Conception Rate
DPR: Daughter Pregnancy Rate
dEBV: Deregressed Estimated Breeding Values
EBV: Estimated Breeding Values
FSC: First Service Conception
GO: Gene Ontology
GWAS: Genome-wide Association Studies
GS: Genomic Selection
GRM: Genomic-based Relationship Matrix
IFLI: Interval From First to Last Insemination
IFEC: Interval to First Estrus After Calving
KEGG: Kyoto Encyclopedia of Genes and Genomes
LD: Linkage Disequilibrium
lncRNA: Long non-coding RNAs
LA: Luteal Activity
MHC: Major Histocompatibility Complex
MAF: Minor Allele Frequency
NRR: Non-return Rate
IPC: Number of Inseminations Per Conception
QC: Quality Controls
QTL: Quantitative Trait Loci
SD: Standard Deviation
SNP: Single-nucleotide Polymorphism
WGS: Whole-genome Sequence
