# Supplementary material for: Identifying pleiotropic variants and candidate genes for fertility and reproduction traits in Holstein cattle via association studies based on imputed whole-genome sequence genotypes
Source: BMC Genomics. 2022 Apr 28;23:331. doi: 10.1186/s12864-022-08555-z (PMC9052698; doi:10.1186/s12864-022-08555-z)
Supplement: Supplementary file 1 — Additional file 1: TableS1. Position distribution and minor allele frequencies of SNPs that passed thegenotype quality control; Table S2. Distribution of significant SNPs of single-traitGWAS; Table S3. Numbers of quantitative trait loci revealed by single-traitGWAS and their overlapping among traits; Table S4. The previously reported andreproduction-associated QTL for the significant SNPs revealed by multiple-traitanalysis of six heifer traits; Table S5. The previously reported andreproduction-associated QTL for the significant SNPs revealed by multiple-traitanalysis of nine cow traits; Table S6. The previously reported andreproduction-associated QTL for the significant SNPs revealed by multiple-traitanalysis of four sire traits; Table S7. Significant SNPs and candidate genesfrom multiple-trait analysis of 14 heifers and cows’ traits; Table S8. Thepreviously reported and reproduction-associated QTL for the significant SNPsrevealed by multiple-trait analysis of 14 heifer and cow traits; Table S9. Thesignificantly enriched GO terms and KEGG from multiple-trait analysis of 18heifers, cows, sire traits; Table S10. Significant SNPs and candidate genesfrom multiple-trait analysis of 18 heifers, cows, sire traits; Table S11. Thepreviously reported and reproduction-associated QTL for the significant SNPsrevealed by multiple-trait analysis of 18 heifer, cow, and sire traits; Figure S1. Manhattan plots (left) and Quantile-quantile plots (right) of thesingle-trait GWAS results based on imputed WGS data; Figure S2. Numbers of significant SNPs foreach trait category and their overlaps found by multiple-trait analysis. Figure S3. Significantly enrichedbiological functions of candidate genes revealed by multiple-trait analysis. Figure S4. Potential protein-protein interaction among biologically relevantgenes that were identified for heifer, cow, and sire traits in this study. [file 12864_2022_8555_MOESM1_ESM.docx]

**Table S1. Position distribution and minor allele frequencies of SNPs that passed the genotype quality control**

| **Traits** | **Number of SNPs** | **Pairwise distances (bp)** | | | **MAF (%)** | | |
| --- | --- | --- | --- | --- | --- | --- | --- |
|  |  | **Mean** | **SD** | **Median** | **Mean** | **SD** | **Median** |
| AFS | 5,564,527 | 446 | 1,477 | 141 | 18.73 | 15.56 | 14.83 |
| FSTCh | 5,498,493 | 452 | 1,504 | 142 | 18.72 | 15.55 | 14.81 |
| NRRh | 5,516,195 | 450 | 1,501 | 142 | 18.70 | 15.56 | 14.77 |
| CEh | 5,670,883 | 438 | 1,439 | 140 | 18.72 | 15.56 | 14.76 |
| CSh | 5,552,017 | 447 | 1,484 | 141 | 18.75 | 15.56 | 14.85 |
| CA | 5,396,362 | 460 | 1,523 | 143 | 18.74 | 15.58 | 14.85 |
| CTFS | 5,647,659 | 440 | 1,454 | 140 | 18.72 | 15.56 | 14.77 |
| DO | 5,590,350 | 444 | 1,469 | 141 | 18.74 | 15.56 | 14.84 |
| FSTCc | 5,592,749 | 444 | 1,477 | 141 | 18.74 | 15.56 | 14.83 |
| NRRc | 5,537,989 | 448 | 1,490 | 141 | 18.76 | 15.57 | 14.89 |
| CEc | 5,640,413 | 440 | 1,464 | 140 | 18.73 | 15.56 | 14.80 |
| CSc | 5,595,112 | 444 | 1,475 | 141 | 18.66 | 15.54 | 14.73 |
| DCA | 5,555,586 | 447 | 1,486 | 141 | 18.72 | 15.56 | 14.81 |
| DF | 5,488,431 | 452 | 1,499 | 142 | 18.74 | 15.57 | 14.87 |
| SCEh | 5,437,403 | 457 | 1,512 | 143 | 18.74 | 15.58 | 14.85 |
| SCEc | 5,558,461 | 447 | 1,467 | 141 | 18.71 | 15.57 | 14.78 |
| SCSh | 5,661,165 | 439 | 1,466 | 140 | 18.64 | 15.53 | 14.66 |
| SCSc | 5,880,012 | 422 | 1,413 | 137 | 18.61 | 15.48 | 14.58 |
| **Average** | **5,576,878** | **445** | **1,478** | **141** | **18.72** | **15.56** | **14.79** |

AFS = Age at first service; FSTCh = First service to conception heifer; NRRh = Non-return rate heifer; CEh = Calving ease heifer; CSh = Calf survival heifer; CA = Calving ability; CTFS =Calving to first service; DO = Days open; FSTCc = First service to conception cow; NRRc = Non-return rate cow; CEc = Calving ease cow; CSc = Calf survival cow; DCA = Daughter calving ability; DF = Daughter fertility; SCEh = Sire calving ease heifer; SCEc = Sire calving ease cow; SCSh = Sire calf survival heifer; SCSc = Sire calf survival cow; MAF = minor allele frequencies.

**Table S2. Distribution of significant SNPs of single-trait GWAS**

| **Chr** | **AFS** | **FSTCh** | **NRRh** | **CEh** | **CSh** | **CA** | **CTFS** | **DO** | **FSTCc** | **NRRc** | **CEc** | **CSc** | **DCA** | **DF** | **SCEc** | **SCEh** | **SCSc** | **SCSh** | **Total** |
| --- | --- | --- | --- | --- | --- | --- | --- | --- | --- | --- | --- | --- | --- | --- | --- | --- | --- | --- | --- |
| BTA1 | 2 | 0 | 2 | 1 | 1 | 2 | 0 | 0 | 1 | 4 | 2 | 2 | 1 | 3 | 0 | 2 | 0 | 4 | 27 |
| BTA2 | 14 | 0 | 4 | 7 | 1 | 1 | 1 | 3 | 1 | 0 | 2 | 2 | 2 | 3 | 0 | 0 | 1 | 5 | 47 |
| BTA3 | 0 | 7 | 0 | 1 | 4 | 0 | 1 | 85 | 0 | 2 | 1 | 2 | 1 | 0 | 5 | 3 | 1 | 1 | 114 |
| BTA4 | 1 | 2 | 2 | 3 | 2 | 0 | 3 | 6 | 0 | 2 | 0 | 6 | 31 | 0 | 7 | 2 | 2 | 1 | 70 |
| BTA5 | 2 | 0 | 0 | 1 | 5 | 1 | 1 | 0 | 3 | 0 | 1 | 1 | 1 | 1 | 22 | 3 | 0 | 0 | 42 |
| BTA6 | 2 | 1 | 2 | 1 | 3 | 1 | 4 | 5 | 4 | 1 | 3 | 5 | 0 | 4 | 0 | 2 | 2 | 2 | 42 |
| BTA7 | 2 | 1 | 1 | 3 | 0 | 4 | 1 | 1 | 4 | 10 | 1 | 0 | 2 | 0 | 5 | 0 | 12 | 0 | 47 |
| BTA8 | 1 | 1 | 3 | 2 | 2 | 15 | 0 | 0 | 1 | 16 | 0 | 12 | 5 | 5 | 1 | 1 | 0 | 1 | 66 |
| BTA9 | 2 | 3 | 0 | 3 | 8 | 2 | 0 | 15 | 1 | 1 | 1 | 2 | 1 | 1 | 2 | 1 | 2 | 7 | 52 |
| BTA10 | 7 | 0 | 2 | 2 | 0 | 4 | 1 | 3 | 2 | 3 | 10 | 8 | 2 | 7 | 6 | 2 | 4 | 0 | 63 |
| BTA11 | 1 | 2 | 1 | 3 | 1 | 1 | 3 | 0 | 1 | 0 | 2 | 1 | 2 | 1 | 5 | 0 | 2 | 1 | 27 |
| BTA12 | 0 | 0 | 1 | 25 | 9 | 1 | 0 | 2 | 1 | 2 | 2 | 1 | 2 | 5 | 4 | 2 | 1 | 2 | 60 |
| BTA13 | 0 | 1 | 0 | 0 | 1 | 4 | 3 | 5 | 1 | 1 | 1 | 0 | 0 | 1 | 1 | 2 | 1 | 0 | 22 |
| BTA14 | 1 | 7 | 2 | 1 | 7 | 4 | 3 | 1 | 3 | 0 | 2 | 0 | 3 | 1 | 2 | 5 | 1 | 1 | 44 |
| BTA15 | 1 | 1 | 0 | 5 | 3 | 1 | 4 | 1 | 2 | 1 | 5 | 4 | 2 | 15 | 0 | 1 | 0 | 2 | 48 |
| BTA16 | 0 | 1 | 0 | 1 | 0 | 0 | 0 | 0 | 1 | 5 | 1 | 1 | 7 | 1 | 2 | 0 | 2 | 1 | 23 |
| BTA17 | 2 | 4 | 0 | 1 | 0 | 2 | 3 | 6 | 3 | 1 | 1 | 0 | 8 | 5 | 5 | 9 | 5 | 1 | 56 |
| BTA18 | 24 | 1 | 2 | 3 | 4 | 1 | 6 | 0 | 0 | 2 | 2 | 2 | 2 | 1 | 2 | 4 | 4 | 2 | 62 |
| BTA19 | 4 | 1 | 2 | 0 | 3 | 6 | 1 | 5 | 3 | 0 | 1 | 3 | 1 | 3 | 2 | 2 | 0 | 2 | 39 |
| BTA20 | 3 | 2 | 4 | 1 | 16 | 4 | 0 | 0 | 2 | 2 | 1 | 4 | 2 | 9 | 2 | 3 | 0 | 14 | 69 |
| BTA21 | 2 | 1 | 0 | 4 | 3 | 4 | 1 | 4 | 3 | 2 | 2 | 0 | 3 | 1 | 1 | 2 | 2 | 0 | 35 |
| BTA22 | 2 | 14 | 2 | 9 | 2 | 13 | 0 | 0 | 3 | 0 | 0 | 1 | 2 | 2 | 7 | 11 | 3 | 4 | 75 |
| BTA23 | 16 | 6 | 2 | 0 | 35 | 3 | 0 | 0 | 0 | 3 | 0 | 4 | 1 | 0 | 1 | 1 | 0 | 0 | 72 |
| BTA24 | 0 | 1 | 1 | 3 | 0 | 6 | 2 | 2 | 1 | 1 | 2 | 1 | 2 | 1 | 4 | 1 | 3 | 11 | 42 |
| BTA25 | 4 | 4 | 0 | 0 | 1 | 3 | 13 | 1 | 5 | 6 | 1 | 2 | 3 | 0 | 1 | 4 | 3 | 0 | 51 |
| BTA26 | 1 | 1 | 3 | 2 | 0 | 4 | 2 | 1 | 1 | 1 | 0 | 2 | 2 | 1 | 1 | 1 | 3 | 14 | 40 |
| BTA27 | 1 | 10 | 0 | 3 | 1 | 1 | 1 | 6 | 4 | 0 | 7 | 1 | 0 | 8 | 1 | 0 | 2 | 3 | 49 |
| BTA28 | 1 | 1 | 9 | 2 | 0 | 0 | 3 | 1 | 2 | 4 | 5 | 1 | 3 | 3 | 9 | 1 | 2 | 1 | 48 |
| BTA29 | 3 | 0 | 1 | 0 | 1 | 1 | 12 | 3 | 0 | 5 | 2 | 0 | 1 | 14 | 1 | 2 | 5 | 1 | 52 |
| **Total** | 99 | 73 | 46 | 87 | 113 | 89 | 69 | 156 | 53 | 75 | 58 | 68 | 92 | 96 | 99 | 67 | 63 | 81 | 1,484 |

AFS = Age at first service; FSTCh = First service to conception heifer; NRRh = Non-return rate heifer; CEh = Calving ease heifer; CSh = Calf survival heifer; CA = Calving ability; CTFS =Calving to first service; DO = Days open; FSTCc = First service to conception cow; NRRc = Non-return rate cow; CEc = Calving ease cow; CSc = Calf survival cow; DCA = Daughter calving ability; DF = Daughter fertility; SCEh = Sire calving ease heifer; SCEc = Sire calving ease cow; SCSh = Sire calf survival heifer; SCSc = Sire calf survival cow; MAF = minor allele frequency.

**Table S3. Numbers of quantitative trait loci revealed by single-trait GWAS and their overlapping among traits**

| **Traits** | **AFS** | **FSTCh** | **NRRh** | **CEh** | **CSh** | **CA** | **CTFS** | **DO** | **FSTCc** | **NRRc** | **CEc** | **CSc** | **DCA** | **DF** | **SCEh** | **SCEc** | **SCSh** | **SCSc** |
| --- | --- | --- | --- | --- | --- | --- | --- | --- | --- | --- | --- | --- | --- | --- | --- | --- | --- | --- |
| **AFS** | **57** | 1 | 1 | 0 | 2 | 2 | 0 | 1 | 2 | 0 | 2 | 1 | 0 | 0 | 0 | 0 | 1 | 0 |
| **FSTCh** |  | **59** | 0 | 1 | 1 | 0 | 1 | 1 | 2 | 0 | 1 | 1 | 0 | 0 | 0 | 1 | 0 | 1 |
| **NRRh** |  |  | **38** | 1 | 1 | 0 | 0 | 0 | 0 | 1 | 0 | 0 | 0 | 1 | 0 | 0 | 0 | 0 |
| **CEh** |  |  |  | **56** | 0 | 0 | 2 | 0 | 0 | 0 | 1 | 0 | 0 | 1 | 0 | 1 | 0 | 0 |
| **CSh** |  |  |  |  | **66** | 1 | 0 | 0 | 0 | 2 | 0 | 2 | 0 | 0 | 1 | 3 | 0 | 2 |
| **CA** |  |  |  |  |  | **57** | 3 | 1 | 1 | 0 | 0 | 0 | 0 | 1 | 1 | 2 | 1 | 1 |
| **CTFS** |  |  |  |  |  |  | **48** | 1 | 1 | 0 | 1 | 1 | 0 | 0 | 1 | 1 | 0 | 0 |
| **DO** |  |  |  |  |  |  |  | **52** | 2 | 0 | 2 | 0 | 2 | 1 | 1 | 0 | 0 | 1 |
| **FSTCc** |  |  |  |  |  |  |  |  | **44** | 0 | 1 | 0 | 0 | 2 | 1 | 1 | 0 | 1 |
| **NRRc** |  |  |  |  |  |  |  |  |  | **47** | 1 | 1 | 1 | 0 | 0 | 1 | 0 | 0 |
| **CEc** |  |  |  |  |  |  |  |  |  |  | **48** | 1 | 1 | 2 | 0 | 0 | 0 | 1 |
| **CSc** |  |  |  |  |  |  |  |  |  |  |  | **57** | 1 | 2 | 0 | 0 | 0 | 0 |
| **DCA** |  |  |  |  |  |  |  |  |  |  |  |  | **54** | 2 | 0 | 2 | 2 | 0 |
| **DF** |  |  |  |  |  |  |  |  |  |  |  |  |  | **57** | 1 | 2 | 1 | 0 |
| **SCEh** |  |  |  |  |  |  |  |  |  |  |  |  |  |  | **52** | 1 | 0 | 0 |
| **SCEc** |  |  |  |  |  |  |  |  |  |  |  |  |  |  |  | **73** | 0 | 0 |
| **SCSh** |  |  |  |  |  |  |  |  |  |  |  |  |  |  |  |  | **54** | 1 |
| **SCSc** |  |  |  |  |  |  |  |  |  |  |  |  |  |  |  |  |  | **54** |

AFS = Age at first service; FSTCh = First service to conception heifer; NRRh = Non-return rate heifer; CEh = Calving ease heifer; CSh = Calf survival heifer; CA = Calving ability; CTFS =Calving to first service; DO = Days open; FSTCc = First service to conception cow; NRRc = Non-return rate cow; CEc = Calving ease cow; CSc = Calf survival cow; DCA = Daughter calving ability; DF = Daughter fertility; SCEh = Sire calving ease heifer; SCEc = Sire calving ease cow; SCSh = Sire calf survival heifer; SCSc = Sire calf survival cow; MAF = minor allele frequency.

**Table S4. The previously reported and reproduction-associated QTL for the significant SNPs revealed by multiple-trait analysis of six heifer traits**

| **SNPs** | | **Known QTL**^1^ | |
| --- | --- | --- | --- |
| **Chromosome** | **Position (bp)** | **ID** | **Name** |
| BTA3 | 89878033 | 120488 | Luteal activity |
|  | 89878033 | 120556 | Luteal activity |
|  | 89878033 | 121399 | Luteal activity |
|  | 89878033 | 176954 | Conception rate |
|  | 89878033 | 121359 | Luteal activity |
|  | 89878033 | 120522 | Luteal activity |
|  | 89878033 | 120516 | Luteal activity |
|  | 89878033 | 120554 | Luteal activity |
|  | 89878033 | 120843 | Luteal activity |
|  | 89878033 | 120495 | Luteal activity |
|  | 89878033 | 120603 | Luteal activity |
|  | 89878033 | 120515 | Luteal activity |
|  | 89878033 | 120594 | Luteal activity |
|  | 89878033 | 120474 | Luteal activity |
|  | 89878033 | 120496 | Luteal activity |
|  | 89878033 | 120502 | Luteal activity |
|  | 89878033 | 120510 | Luteal activity |
|  | 89878033 | 120519 | Luteal activity |
|  | 89878033 | 120527 | Luteal activity |
|  | 89878033 | 120535 | Luteal activity |
|  | 89878033 | 120542 | Luteal activity |
|  | 89878033 | 120553 | Luteal activity |
|  | 89878033 | 120564 | Luteal activity |
|  | 89878033 | 120570 | Luteal activity |
|  | 89878033 | 120575 | Luteal activity |
|  | 89878033 | 120604 | Luteal activity |
|  | 89878033 | 120639 | Luteal activity |
|  | 89878033 | 121364 | Luteal activity |
|  | 89878033 | 121365 | Luteal activity |
|  | 89878033 | 121366 | Luteal activity |
|  | 89878033 | 121367 | Luteal activity |
|  | 89878033 | 121368 | Luteal activity |
|  | 89878033 | 121369 | Luteal activity |
|  | 89878033 | 121370 | Luteal activity |
|  | 89878033 | 121371 | Luteal activity |
|  | 89878033 | 121372 | Luteal activity |
|  | 89878033 | 121373 | Luteal activity |
|  | 89878033 | 121374 | Luteal activity |
|  | 89878033 | 121375 | Luteal activity |
|  | 89878033 | 121376 | Luteal activity |
|  | 89878033 | 121377 | Luteal activity |
|  | 89878033 | 121378 | Luteal activity |
|  | 89878033 | 121379 | Luteal activity |
|  | 89878033 | 121380 | Luteal activity |
|  | 89878033 | 121381 | Luteal activity |
|  | 89878033 | 121382 | Luteal activity |
|  | 89878033 | 121383 | Luteal activity |
|  | 89878033 | 121384 | Luteal activity |
|  | 89878033 | 121385 | Luteal activity |
|  | 89878033 | 121387 | Luteal activity |
|  | 89878033 | 121388 | Luteal activity |
|  | 89878033 | 121389 | Luteal activity |
|  | 89878033 | 121390 | Luteal activity |
|  | 89878033 | 121391 | Luteal activity |
|  | 89878033 | 121392 | Luteal activity |
|  | 89878033 | 121393 | Luteal activity |
|  | 89878033 | 121394 | Luteal activity |
|  | 89878033 | 121395 | Luteal activity |
|  | 89878033 | 121396 | Luteal activity |
|  | 89878033 | 121397 | Luteal activity |
|  | 89878033 | 121398 | Luteal activity |
|  | 89878033 | 121400 | Luteal activity |
|  | 89878033 | 121401 | Luteal activity |
|  | 89878033 | 121402 | Luteal activity |
|  | 89878033 | 121403 | Luteal activity |
|  | 89878033 | 121404 | Luteal activity |
|  | 89878033 | 121405 | Luteal activity |
|  | 89878033 | 121406 | Luteal activity |
|  | 89878033 | 121407 | Luteal activity |
|  | 89878033 | 121410 | Luteal activity |
|  | 89878033 | 121411 | Luteal activity |
|  | 89878033 | 120947 | Luteal activity |
|  | 89878033 | 120487 | Luteal activity |
|  | 89878033 | 120490 | Luteal activity |
|  | 89878033 | 120499 | Luteal activity |
|  | 89878033 | 120520 | Luteal activity |
|  | 89878033 | 120528 | Luteal activity |
|  | 89878033 | 120566 | Luteal activity |
|  | 89878033 | 120577 | Luteal activity |
|  | 89878033 | 120598 | Luteal activity |
|  | 89878033 | 120606 | Luteal activity |
|  | 89878033 | 120608 | Luteal activity |
|  | 89878033 | 120618 | Luteal activity |
|  | 89878033 | 120624 | Luteal activity |
|  | 89878033 | 120637 | Luteal activity |
|  | 89878033 | 120444 | Luteal activity |
|  | 89878033 | 121350 | Luteal activity |
|  | 89878033 | 121352 | Luteal activity |
|  | 89878033 | 121353 | Luteal activity |
|  | 89878033 | 121354 | Luteal activity |
|  | 89878033 | 121331 | Luteal activity |
|  | 89878033 | 121347 | Luteal activity |
|  | 89878033 | 120394 | Luteal activity |
|  | 89878033 | 120642 | Luteal activity |
|  | 89878033 | 121355 | Luteal activity |
|  | 89878033 | 121356 | Luteal activity |
|  | 89878033 | 121358 | Luteal activity |
|  | 89878033 | 120658 | Luteal activity |
| BTA6 | 31807526 | 30366 | Interval to first estrus after calving |
|  | 31807526 | 30119 | Interval to first estrus after calving |
|  | 54108303 | 42006 | Calving ease maternal |
| BTA7 | 6177026 | 42676 | Stillbirth maternal |
|  | 100189059 | 29910 | Age at puberty |
|  | 100189059 | 29905 | Age at puberty |
| BTA9 | 95975870 | 122349 | Daughter pregnancy rate |
|  | 95975870 | 122378 | Conception rate |
|  | 95975870 | 57096 | Daughter pregnancy rate |
|  | 95975870 | 57145 | Conception rate |
|  | 95975870 | 30279 | Interval to first estrus after calving |
| BTA10 | 81606975 | 44961 | Calving ease |
| BTA12 | 77682412 | 24604 | Calving ease |
|  | 77682412 | 46533 | Daughter pregnancy rate |
|  | 77682412 | 46542 | Calving ease |
|  | 77682412 | 46543 | Stillbirth |
| BTA14 | 18883604 | 212276 | First service conception |
|  | 18883604 | 212774 | Inseminations per conception |
|  | 18883604 | 181313 | Conception rate |
|  | 54326109 | 177153 | Conception rate |
|  | 61221242 | 181510 | Inseminations per conception |
|  | 61221242 | 181318 | Conception rate |
|  | 61221242 | 106988 | Daughter pregnancy rate |
| BTA18 | 41105349 | 212858 | Inseminations per conception |
|  | 41112039 | 212858 | Inseminations per conception |
| BTA19 | 46856509 | 212874 | Inseminations per conception |
|  | 46856509 | 212329 | First service conception |
| BTA22 | 5159344 | 121667 | Calving interval |
|  | 51337027 | 106882 | Conception rate |
|  | 51337027 | 106883 | Conception rate |
|  | 55900928 | 51841 | Calving ease maternal |
| BTA23 | 28036775 | 172223 | Age at puberty |
| BTA25 | 38933869 | 121698 | Calving interval |
|  | 38933869 | 52564 | Daughter pregnancy rate |
|  | 38933869 | 52569 | Calving ease |
| BTA27 | 13585281 | 177292 | Conception rate |
|  | 34758502 | 53537 | Calving ease maternal |
|  | 34758502 | 53545 | Calving ease |
|  | 34758502 | 53546 | Stillbirth |
| BTA28 | 10602903 | 177304 | Conception rate |

^1^ All references related to these QTL were linked to QTL ID and could be found at https://www.animalgenome.org/cgi-bin/QTLdb/BT/index.

**Table S5. The previously reported and reproduction-associated QTL for the significant SNPs revealed by multiple-trait analysis of nine cow traits**

| **SNPs** | | **Known QTL**^1^ | |
| --- | --- | --- | --- |
| **Chromosome** | **Position bp** | **ID** | **Name** |
| BTA5 | 59741942 | 71280 | Inhibin level |
|  | 61923964 | 176714 | First service conception |
|  | 61923964 | 176989 | Conception rate |
| BTA9 | 91316160 | 44287 | Stillbirth maternal |
| BTA10 | 15091095 | 212225 | First service conception |
|  | 15091095 | 212688 | Inseminations per conception |
| BTA11 | 89383411 | 46045 | Calving ease maternal |
|  | 89383411 | 46056 | Calving ease |
|  | 89383411 | 46057 | Stillbirth |
| BTA12 | 74922982 | 176794 | First service conception |
|  | 74922982 | 177135 | Conception rate |
| BTA14 | 2204649 | 15043 | Stillbirth |
| BTA15 | 54978763 | 107149 | Conception rate |
| BTA17 | 4778666 | 212828 | Inseminations per conception |
|  | 36481658 | 212839 | Inseminations per conception |
| BTA18 | 63182112 | 101376 | Calving interval |
|  | 63182112 | 101379 | Calving interval |
|  | 63182112 | 101381 | Calving interval |
|  | 63182112 | 101382 | Calving interval |
|  | 63182112 | 101380 | Calving interval |
|  | 63182112 | 147053 | Stillbirth |
| BTA20 | 56959618 | 177248 | Conception rate |
|  | 56959618 | 176845 | First service conception |
|  | 56959618 | 212337 | First service conception |
|  | 56959618 | 212895 | Inseminations per conception |
| BTA24 | 57019331 | 212962 | Inseminations per conception |
| BTA25 | 27413783 | 64974 | Age at first calving |
| BTA27 | 24754025 | 62078 | Sperm motility |
|  | 43998949 | 121670 | Calving interval |
| BTA 29 | 37010381 | 57125 | Daughter pregnancy rate |
|  | 37010381 | 57142 | Conception rate |

^1^ All references related to these QTL were linked to QTL ID and could be found at https://www.animalgenome.org/cgi-bin/QTLdb/BT/index.

**Table S6. The previously reported and reproduction-associated QTL for the significant SNPs revealed by multiple-trait analysis of four sire traits**

| **SNPs** | | **Known QTL**^1^ | |
| --- | --- | --- | --- |
| **Chromosome** | **Position bp** | **ID** | **Name** |
| BTA4 | 82878864 | 40565 | Calving ease maternal |
|  | 82878864 | 40577 | Calving ease |
|  | 82878864 | 40578 | Stillbirth |
| BTA5 | 53516007 | 138995 | Scrotal circumference |
|  | 53516007 | 138996 | Scrotal circumference |
|  | 53516007 | 138997 | Scrotal circumference |
|  | 53516007 | 138999 | Scrotal circumference |
|  | 53516007 | 71506 | Inhibin level |
|  | 53516007 | 138998 | Scrotal circumference |
| BTA6 | 7494351 | 181464 | Inseminations per conception |
|  | 7494351 | 181217 | Conception rate |
| BTA7 | 64117199 | 212619 | Inseminations per conception |
|  | 64117199 | 125307 | Twinning |
|  | 83320343 | 107102 | Conception rate |
|  | 100056219 | 29910 | Age at puberty |
|  | 100056219 | 29905 | Age at puberty |
| BTA9 | 15828286 | 177070 | Conception rate |
|  | 15843031 | 177070 | Conception rate |
|  | 74655065 | 169800 | Sexual precocity |
|  | 74655065 | 181491 | Inseminations per conception |
|  | 74655065 | 181260 | Conception rate |
|  | 74655065 | 107117 | Conception rate |
| BTA13 | 53595922 | 181307 | Conception rate |
|  | 53595922 | 181504 | Inseminations per conception |
| BTA 15 | 30636699 | 30177 | Interval to first estrus after calving |
|  | 30636699 | 29787 | Age at puberty |
|  | 30636699 | 30428 | Interval to first estrus after calving |
|  | 60970873 | 212802 | Inseminations per conception |
|  | 60970873 | 212292 | First service conception |
| BTA 17 | 18408888 | 107000 | Daughter pregnancy rate |
|  | 18458616 | 107000 | Daughter pregnancy rate |
|  | 18458616 | 107001 | Daughter pregnancy rate |
|  | 33971794 | 212838 | Inseminations per conception |
|  | 33971794 | 49204 | Calving ease maternal |
|  | 33971794 | 49205 | Daughter pregnancy rate |
|  | 33971794 | 49218 | Calving ease |
|  | 33971794 | 49220 | Stillbirth |
|  | 48501570 | 30195 | Interval to first estrus after calving |
|  | 48501570 | 30413 | Interval to first estrus after calving |
|  | 73040719 | 181526 | Inseminations per conception |
|  | 73040719 | 181354 | Conception rate |
|  | 73040719 | 177211 | Conception rate |
|  | 73040719 | 176830 | First service conception |
|  | 73040719 | 140628 | Non-return rate |
|  | 73040719 | 142307 | Non-return rate |
|  | 73040719 | 122410 | Conception rate |
|  | 73040719 | 57101 | Daughter pregnancy rate |
| BTA19 | 23803632 | 30204 | Interval to first estrus after calving |
|  | 23811867 | 30204 | Interval to first estrus after calving |
| BTA22 | 37660216 | 51659 | Calving ease maternal |
|  | 37660216 | 51660 | Daughter pregnancy rate |
|  | 37660216 | 51661 | Stillbirth maternal |
|  | 42471770 | 51746 | Daughter pregnancy rate |
|  | 42471770 | 51751 | Daughter pregnancy rate |
|  | 42471770 | 51756 | Calving ease |
| BTA24 | 20595336 | 31049 | Calving interval |
|  | 61476879 | 107040 | Daughter pregnancy rate |
| BTA25 | 26121115 | 64973 | Age at first calving |
|  | 27554453 | 64974 | Age at first calving |
|  | 39147647 | 36564 | Conception rate |
| BTA26 | 10927671 | 29950 | Age at puberty |
|  | 48212324 | 177290 | Conception rate |
|  | 48212324 | 53192 | Calving ease maternal |
|  | 48212324 | 53194 | Daughter pregnancy rate |
|  | 48212324 | 53207 | Calving ease |
|  | 48212324 | 53209 | Stillbirth |
| BTA27 | 13351474 | 63720 | Reproductive efficiency |
|  | 25506261 | 177297 | Conception rate |
| BTA 28 | 5066852 | 177302 | Conception rate |
|  | 37896374 | 53812 | Calving ease |
| BTA29 | 4807970 | 106902 | Conception rate |
|  | 14612904 | 177315 | Conception rate |

^1^ All references related to these QTL were linked to QTL ID and could be found at https://www.animalgenome.org/cgi-bin/QTLdb/BT/index.

**Table S7. Significant SNPs and candidate genes from multiple-trait analysis of 14 heifers and cows traits**

| **Chr** | **Position (bp)** | ***P* values** | **Location** | **Candidate genes**  **(within ± 100 kb)^1^** | **N of QTL^2^** |
| --- | --- | --- | --- | --- | --- |
| BTA1 | 72,998,070 | 5.193e-06 | Intergenic | *LSG1*, *TMEM44*, *ATP13A3* | 0 |
|  | 102,173,402 | 1.5662e-05 | Intergenic | *SLITRK3*, *SI* | 0 |
|  | 154,266,412 | 1.966e-05 | Intron | ***TBC1D5*** | 0 |
| BTA2 | 60,828,766 | 1.3641e-05 | Intergenic | None | 0 |
|  | 70,633,250 | 6.3701e-06 | Intergenic | *EN1* | 0 |
|  | 88,926,474 | 7.3981e-06 | Intron | ***SPATS2L*** | 0 |
| BTA3 | 67,691,415 | 1.0173e-05 | Intron | ***ST6GAL0C5*** | 0 |
| BTA4 | 14,828,190 | 2.2285e-05 | Intergenic | None | 0 |
|  | 17,304,670 | 1.5736e-05 | Intergenic | None | 0 |
|  | 56,643,360 | 7.583e-07 | Intergenic | *DOCK4* | 0 |
|  | 86,337,952 | 1.9706e-06 | Intron | ***PTPRZ1*** | 0 |
|  | 86,338,437 | 1.4297e-06 | Intron |  |  |
|  | 89,601,703 | 2.8272e-05 | Intergenic | None | 0 |
|  | 89,602,920 | 2.5802e-05 | Intergenic |  |  |
|  | 89,604,363 | 1.5907e-05 | Intergenic |  |  |
|  | 89,674,420 | 2.6111e-05 | Intergenic |  |  |
| BTA5 | 15,850,823 | 2.0093e-05 | Intergenic | None | 0 |
| BTA6 | 54,108,303 | 2.0442e-05 | Intergenic | None | 1 |
|  | 93,858,468 | 7.2232e-06 | Intergenic | None | 5 |
| BTA7 | 62,865,717 | 2.9957e-05 | Intergenic | *FAT2*, *SPARC*, ENSBTAG00000054940, *ATOX1* | 0 |
|  | 100,189,059 | 2.2799e-05 | Intergenic | *ST8SIA4* | 2 |
| BTA8 | 6,524,101 | 2.6493e-05 | Intergenic | ENSBTAG00000052081*, *FBXO8*, *CEP44* | 0 |
|  | 48,903,003 | 1.2833e-05 | Intergenic | ENSBTAG00000048940*, *TMC1*, ENSBTAG00000052698 | 0 |
|  | 49,280,626 | 7.5476e-06 | Intergenic | ENSBTAG00000052764*, *ANXA1* | 0 |
|  | 49,282,386 | 8.7628e-06 | Intergenic |  |  |
|  | 49,282,702 | 2.2652e-06 | Intergenic |  |  |
|  | 60,191,474 | 2.5704e-05 | Upstream | *OR13J1F*, *OR13J1*, *OR13J1D*, *OR13J1G*, *OR13E12*, ***OR13J1B***, *HRCT1*, *SPAAR*, *OR13C11*, *OR13C7*, *OR13C7L*, *OR13C7B* | 1 |
|  | 97,982,105 | 9.6495e-06 | Intergenic | None | 0 |
|  | 98,009,209 | 7.9918e-06 | Intergenic |  |  |
|  | 98,978,292 | 3.7751e-06 | Intron | ***PTPN3*** | 0 |
|  | 98,998,669 | 1.2891e-06 | Intron |  |  |
|  | 99,035,466 | 1.1489e-05 | Intron |  |  |
| BTA9 | 5,744,574 | 2.1698e-05 | Intergenic | None | 4 |
|  | 21,350,462 | 1.0121e-05 | Intergenic | None | 0 |
|  | 70,186,779 | 2.9434e-05 | Intergenic | ENSBTAG00000053423, *MOXD1* | 1 |
|  | 71,191,104 | 1.1016e-05 | Intergenic | None | 0 |
|  | 77,009,122 | 2.4627e-05 | Intergenic | *TXLNB*, *CITED2* | 0 |
|  | 77,013,726 | 2.7033e-05 | Intergenic |  |  |
|  | 82,830,918 | 3.1632e-05 | Intergenic | *EPM2A* | 0 |
| BTA10 | 15,737,285 | 7.2104e-06 | Intergenic | *ANP32A*, ENSBTAG00000051138*, *SPESP1* | 0 |
|  | 27,718,962 | 1.2059e-05 | Intergenic | *OR4F67B*, *OR4G18*, *OR4K36*, *OR4G8*, *OR4G9*, *OR4G2* | 0 |
|  | 33,760,361 | 1.718e-05 | Intergenic | *SPRED1* | 1 |
|  | 34,575,527 | 2.3976e-05 | Intergenic | None | 4 |
|  | 39,839,364 | 2.8463e-05 | Intron | **ENSBTAG00000054557** | 1 |
| BTA11 | 6,323,341 | 2.4129e-05 | Intron | *CREG2*, ***RFX8*** | 0 |
| BTA12 | 37,729,885 | 1.1747e-05 | Intergenic | None | 0 |
| BTA13 | 20,203,933 | 1.1564e-06 | Intergenic | ENSBTAG00000002675, ENSBTAG00000052951 | 0 |
|  | 35,506,150 | 9.7887e-06 | Intergenic | ENSBTAG00000055307*, *LYZL1* | 3 |
| BTA14 | 18,883,604 | 1.7281e-05 | Intergenic | None | 3 |
|  | 43,546,785 | 2.9039e-05 | Intergenic | None | 12 |
|  | 79,535,150 | 1.5974e-05 | Intergenic | None | 0 |
|  | 79,845,199 | 3.5598e-05 | Intergenic | None | 0 |
| BTA15 | 54,978,763 | 3.4705e-05 | Intergenic | *MOGAT2*, ENSBTAG00000053794, ENSBTAG00000047111, ENSBTAG00000017443, ENSBTAG00000015091, ENSBTAG00000052936 | 1 |
| BTA16 | 75,886,717 | 3.8457e-05 | Intron | ENSBTAG00000050070, ENSBTAG00000053010*, **ENSBTAG00000030910**, ENSBTAG00000048618, *F13B*, *CFHR5*, ***ASPM***, *ZBTB41* | 3 |
|  | 76,071,433 | 1.8661e-05 | Intron |  |  |
| BTA17 | 4,778,666 | 8.7408e-06 | Intron | *FHDC1*, ***ARFIP1*** | 1 |
|  | 32,848,516 | 1.5961e-05 | Intergenic | None | 0 |
| BTA18 | 35,987,322 | 2.8639e-06 | Intron | *ZFP90*, ENSBTAG00000053442, ***CDH3***, *CDH1* | 0 |
|  | 39,750,065 | 4.7346e-05 | Intergenic | ENSBTAG00000053994*, *CALB2*, *CMTR2*, ENSBTAG00000051512 | 0 |
|  | 39,797,494 | 6.5442e-06 | Intergenic |  |  |
|  | 61,968,892 | 4.2152e-05 | Intergenic | *EPN1*, *U2AF2*, *CCDC106*, *ZNF581*, *ZNF580*, *ZNF524*, *ZNF784*, *FIZ1*, *ZNF579*, *SBK2,* *SSC5D*, *0T14*, *ZNF628*, *C19orf85*, ENSBTAG00000050011, *ISOC2*, *SHISA7* | 0 |
| BTA19 | 53,779,550 | 9.1364e-06 | Intron | ***D0H17***, *PGS1*, *SOCS3* | 0 |
|  | 59,744,349 | 4.769e-06 | Intergenic | None | 0 |
| BTA20 | 2,510,776 | 1.909e-05 | Intron | ***KCNIP1*** | 2 |
|  | 29,957,655 | 3.9937e-05 | Intergenic | None | 0 |
| BTA21 | 4,494,184 | 4.338e-05 | Intergenic | ENSBTAG00000051536 | 0 |
|  | 10,251,629 | 2.4075e-05 | Intergenic | None | 0 |
|  | 37,955,453 | 4.26e-05 | Intergenic | ENSBTAG00000031558 | 1 |
|  | 54,817,687 | 4.305e-05 | Intron | *KLHL28*, *TOGARAM1*, ***PRPF39***, *FKBP3*, *FANCM*, *MIS18BP1* | 1 |
|  | 60,917,828 | 9.0438e-06 | Intron | ***C21H14orf132*** | 0 |
| BTA22 | 5,160,840 | 3.3353e-07 | Intron | ***TGFBR2***, *GADL1* | 1 |
|  | 11,737,948 | 1.4902e-05 | Downstream | *OXSR1*, ENSBTAG000000**50531***, *SLC22A14*, *XYLB*, *ACVR2B* | 0 |
|  | 56,807,677 | 9.8686e-06 | Intron | ***PPARG***, ENSBTAG00000052393* | 0 |
|  | 57,243,658 | 2.4287e-05 | Intron | ENSBTAG00000031115, *RBSN*, ***MRPS25***, *NR2C2* | 2 |
| BTA23 | 1,002,264 | 2.0938e-05 | Intergenic | *KHDRBS2* | 0 |
|  | 2,330,898 | 2.3856e-07 | Intergenic | None | 0 |
|  | 2,333,013 | 3.4912e-05 | Intergenic |  |  |
|  | 2,804,180 | 7.8878e-06 | Intron | ***PRIM2*** | 2 |
|  | 2,823,702 | 5.2843e-06 | Intron |  |  |
|  | 2,825,300 | 5.3385e-05 | Intron |  |  |
|  | 3,241,158 | 4.9829e-05 | Intron | *ZNF451*, ***BEND6*** | 0 |
|  | 3,504,801 | 7.5268e-06 | Intron | **ENSBTAG00000051082** | 0 |
|  | 5,466,948 | 3.6367e-05 | Intron | ***FAM83B*** | 0 |
|  | 17,164,665 | 2.7059e-05 | Downstream | *POLR1C*, *XPO5*, *POLH*, *GTPBP2*, ***MAD2L1BP***, ***RSPH9***, *MRPS18A*, ENSBTAG00000050989* | 5 |
|  | 28,726,064 | 7.3368e-06 | Upstream | *JSP.1*, ENSBTAG00000037421, ***BOLA***, *TRIM26*, *TRIM15*, *TRIM10* | 0 |
| BTA24 | 58,070,140 | 4.7037e-05 | Intron | *ZNF532*, **ENSBTAG00000022829**, *SEC11C* | 0 |
| BTA25 | 8,702,138 | 1.0971e-05 | Intergenic | *GRIN2A* | 0 |
| BTA27 | 24,754,025 | 5.0909e-05 | Intron | *CLDN23*, **ENSBTAG00000053191** | 1 |
|  | 43,998,949 | 6.5867e-06 | Intergenic | *ZNF385D* | 1 |
| BTA28 | 24,802,568 | 7.0867e-06 | Intron | *PBLD*, *HNRNPH3*, ***RUFY2***, *D02*, *SLC25A16* | 0 |
|  | 35,484,133 | 6.2494e-05 | Upstream | *CGN1*, *CL46*, **ENSBTAG00000052810**, *CL43*, ENSBTAG00000048082, *SFTPD*, *MBL1*, *SFTPA1*, ENSBTAG00000052322*, *MAT1A*, *DYDC1* | 0 |
|  | 35,630,767 | 1.0379e-05 | Intergenic |  |  |
|  | 40,034,955 | 1.3056e-06 | Intergenic | None | 0 |
| BTA29 | 3,939,059 | 4.9235e-05 | Intergenic | None | 0 |
|  | 13,401,407 | 5.8712e-05 | Intergenic | None | 1 |
|  | 40,093,997 | 3.335e-05 | Intergenic | *SYT7*, *DAGLA* | 0 |

^1^ Candidate genes are represented by gene symbol when available, otherwise by the Ensembl gene ID. The long noncoding RNA genes are marked by asterisk (*). The genes directly linked to SNPs are further denoted in bold.

^2^ The number of known and reproduction-associated QTL found in Cattle QTL Database v43 (www.animalgenome.org).

**Table S8. The previously reported and reproduction-associated QTL for the significant SNPs revealed by multiple-trait analysis of 14 heifers and cows traits**

| **SNPs** | | **Known QTL** | |
| --- | --- | --- | --- |
| **Chromosome** | **Position bp** | **ID** | **Name** |
| BTA6 | 54108303 | 42006 | Calving ease maternal |
|  | 93858468 | 173429 | Daughter pregnancy rate |
|  | 93858468 | 173430 | Daughter pregnancy rate |
|  | 93858468 | 176402 | Conception rate |
|  | 93858468 | 176403 | Conception rate |
|  | 93858468 | 176404 | Conception rate |
| BTA7 | 100189059 | 29910 | Age at puberty |
|  | 100189059 | 29905 | Age at puberty |
| BTA8 | 60191474 | 212644 | Inseminations per conception |
| BTA9 | 5744574 | 212654 | Inseminations per conception |
|  | 5744574 | 212195 | First service conception |
|  | 5744574 | 176758 | First service conception |
|  | 5744574 | 177066 | Conception rate |
|  | 70186779 | 176408 | Conception rate |
| BTA10 | 33760361 | 101297 | Age at puberty |
|  | 34575527 | 44451 | Calving ease maternal |
|  | 34575527 | 44452 | Daughter pregnancy rate |
|  | 34575527 | 44453 | Stillbirth maternal |
|  | 34575527 | 44458 | Stillbirth |
|  | 39839364 | 176768 | First service conception |
| BTA13 | 35506150 | 212762 | Inseminations per conception |
|  | 35506150 | 30085 | Interval to first estrus after calving |
|  | 35506150 | 30294 | Interval to first estrus after calving |
| BTA14 | 18883604 | 212276 | First service conception |
|  | 18883604 | 212774 | Inseminations per conception |
|  | 18883604 | 181313 | Conception rate |
|  | 43546785 | 212284 | First service conception |
|  | 43546785 | 212784 | Inseminations per conception |
|  | 43546785 | 138861 | Scrotal circumference |
|  | 43546785 | 138862 | Scrotal circumference |
|  | 43546785 | 138863 | Scrotal circumference |
|  | 43546785 | 139083 | Scrotal circumference |
|  | 43546785 | 139084 | Scrotal circumference |
|  | 43546785 | 138865 | Scrotal circumference |
|  | 43546785 | 138866 | Scrotal circumference |
|  | 43546785 | 138867 | Scrotal circumference |
|  | 43546785 | 138868 | Scrotal circumference |
|  | 43546785 | 139085 | Scrotal circumference |
| BTA15 | 54978763 | 107149 | Conception rate |
| BTA16 | 75886717 | 56424 | Calving ease |
|  | 75886717 | 177190 | Conception rate |
|  | 76071433 | 177190 | Conception rate |
| BTA17 | 4778666 | 212828 | Inseminations per conception |
| BTA20 | 2510776 | 50349 | Stillbirth maternal |
|  | 2510776 | 50352 | Calving ease maternal |
| BTA21 | 37955453 | 212907 | Inseminations per conception |
|  | 54817687 | 107029 | Daughter pregnancy rate |
| BTA22 | 5160840 | 121667 | Calving interval |
|  | 57243658 | 107215 | Conception rate |
|  | 57243658 | 107038 | Daughter pregnancy rate |
| BTA23 | 2804180 | 51892 | Stillbirth maternal |
|  | 2804180 | 51903 | Stillbirth maternal |
|  | 2823702 | 51892 | Stillbirth maternal |
|  | 2823702 | 51903 | Stillbirth maternal |
|  | 2825300 | 51892 | Stillbirth maternal |
|  | 2825300 | 51903 | Stillbirth maternal |
|  | 17164665 | 212937 | Inseminations per conception |
|  | 17164665 | 176853 | First service conception |
|  | 17164665 | 177264 | Conception rate |
|  | 17164665 | 52095 | Calving ease maternal |
|  | 17164665 | 52102 | Calving ease |
| BTA27 | 24754025 | 62078 | Sperm motility |
|  | 43998949 | 121670 | Calving interval |
| BTA29 | 13401407 | 181417 | Conception rate |

**Table S9. The significantly enriched GO terms and KEGG from multiple-trait analysis of 18 heifers, cows, sire traits**

| **Trait categories** | **Sources** | **Term names** | ***P* values** |
| --- | --- | --- | --- |
| HC | KEGG | Phagosome | 2.7550e-05 |
|  | GO:CC | Rough endoplasmic reticulum | 4.8063e-05 |
|  | GO:MF | 2-acylglycerol O-acyltransferase activity | 9.2366e-05 |
|  | GO:BP | Diacylglycerol metabolic process | 1.1413e-03 |
|  | GO:BP | Diacylglycerol biosynthetic process | 1.3544e-03 |
|  | GO:CC | Multivesicular body | 1.6254e-03 |
|  | GO:BP | Surfactant homeostasis | 2.9017e-03 |
|  | GO:BP | Chemical homeostasis within a tissue | 5.4776e-03 |
|  | GO:MF | Acylglycerol O-acyltransferase activity | 5.9636e-03 |
|  | GO:CC | Collagen trimer | 6.6715e-03 |
| HCS | GO:MF | 2-acylglycerol O-acyltransferase activity | 0.00017173 |
|  | GO:BP | Diacylglycerol biosynthetic process | 0.00169740 |
|  | GO:MF | Acylglycerol O-acyltransferase activity | 0.01101720 |
|  | GO:MF | O-acyltransferase activity | 0.02843661 |

HC: heifers plus cows; HCS: all the animals together.

**Table S10. Significant SNPs and candidate genes from multiple-trait analysis of 18 heifers, cows, sire traits**

| **Chr** | **Position (bp)** | ***P* values** | **Location** | **Candidate genes**  **(within ± 100 kb)^1^** | **N of QTL^2^** |
| --- | --- | --- | --- | --- | --- |
| BTA1 | 102,173,402 | 1.2403e-05 | Intergenic | *SLITRK3*, *SI* | 0 |
|  | 114,863,175 | 1.6825e-05 | Intergenic | *P2RY1* | 0 |
| BTA2 | 60,828,766 | 2.0721e-05 | Intergenic | None | 0 |
| BTA4 | 56,643,360 | 2.353e-06 | Intergenic | *DOCK4* | 0 |
|  | 86,337,952 | 7.5007e-06 | Intron | ***PTPRZ1*** | 0 |
|  | 86,338,437 | 2.6056e-06 | Intron |  |  |
| BTA5 | 6,106,039 | 1.7583e-05 | Intergenic | *ZDHHC17* | 0 |
|  | 9,837,342 | 2.4143e-05 | Intron | ***OTOGL*** | 1 |
|  | 35,789,584 | 2.6662e-05 | Intron | ***NELL2*** | 0 |
| BTA6 | 93,858,468 | 1.4344e-05 | Intergenic | None | 5 |
| BTA7 | 62,865,717 | 2.4983e-05 | Intergenic | *FAT2*, *SPARC*, ENSBTAG00000054940, *ATOX1* | 0 |
|  | 93,240,361 | 1.4799e-05 | Intron | ***FAM172A*** | 0 |
|  | 96,545,772 | 2.617e-05 | Intron | *LNPEP*, ENSBTAG00000003118, ENSBTAG00000052101*, ***LIX1***, *RIOK2*, ENSBTAG00000054614* | 4 |
|  | 100,189,059 | 1.914e-05 | Intergenic | *ST8SIA4* | 2 |
| BTA8 | 49,282,702 | 1.681e-05 | Intergenic | ENSBTAG00000052764*, *ANXA1* | 0 |
|  | 60,191,474 | 6.6144e-06 | Upstream | *OR13J1F*, *OR13J1*, *OR13J1D*, *OR13J1G*, *OR13E12*, ***OR13J1B***, *HRCT1*, *SPAAR*, *OR13C11*, *OR13C7*, *OR13C7L*, *OR13C7B* | 1 |
|  | 85,035,847 | 2.9359e-05 | Intergenic | *PHF2*, ENSBTAG00000050999, ENSBTAG00000050528 | 5 |
|  | 98,978,292 | 2.5925e-05 | Intron | ***PTPN3*** | 0 |
|  | 98,998,669 | 3.9349e-06 | Intron |  |  |
|  | 99,035,466 | 2.2073e-05 | Intron |  |  |
|  | 99,060,131 | 4.3062e-06 | Intron |  |  |
| BTA9 | 71,063,417 | 2.29e-05 | Intergenic | *SLC18B1*, *RPS12* | 0 |
|  | 71,191,104 | 2.3964e-05 | Intergenic |  |  |
|  | 77,009,122 | 1.7769e-05 | Intergenic | *TXLNB*, *CITED2* | 0 |
|  | 77,013,726 | 1.7776e-06 | Intergenic |  |  |
|  | 82,830,918 | 1.9138e-05 | Intergenic | *EPM2A* | 0 |
| BTA10 | 15,091,095 | 1.8603e-05 | Intergenic | *PIAS1*, *CALML4*, *CLN6*, *FEM1B*, *ITGA11* | 2 |
|  | 15,737,285 | 2.3662e-05 | Intergenic | *ANP32A*, ENSBTAG00000051138*, *SPESP1* | 0 |
|  | 27,718,962 | 2.83e-05 | Intergenic | *OR4F67B*, *OR4G18*, *OR4K36*, *OR4G8*, *OR4G9*, *OR4G2* | 0 |
|  | 39,839,364 | 2.7369e-05 | Intron | **ENSBTAG00000054557** | 1 |
|  | 43,187,675 | 2.366e-05 | Intron | *SOS2*, ***L2HGDH***, *DMAC2L*, *CDKL1* | 2 |
|  | 54,540,458 | 7.6556e-06 | Intron | ***NEDD4***, ENSBTAG00000031396, *PRTG* | 0 |
| BTA12 | 37,729,885 | 3.7374e-05 | Intergenic | None | 0 |
|  | 51,864,428 | 1.3915e-05 | Intergenic | ENSBTAG00000053180* | 4 |
| BTA13 | 20,203,933 | 6.4123e-06 | Intergenic | ENSBTAG00000002675, ENSBTAG00000052951 | 0 |
|  | 35,506,150 | 3.276e-05 | Intergenic | ENSBTAG00000055307*, *LYZL1* | 3 |
|  | 40,206,269 | 1.409e-05 | Intergenic | *RALGAPA2* | 0 |
|  | 41,398,321 | 1.9136e-05 | Intergenic | ENSBTAG00000048400* | 0 |
| BTA14 | 3,467,609 | 3.8292e-05 | Intron | ***TRAPPC9*** | 0 |
|  | 20,411,413 | 1.5009e-05 | Intron | ***SNTG1*** | 0 |
|  | 20,446,256 | 2.5984e-05 | Intron |  |  |
|  | 58,928,413 | 2.9153e-05 | Intron | ***ZFPM2*** | 0 |
|  | 79,535,150 | 3.4301e-05 | Intergenic | None | 0 |
| BTA15 | 54,978,763 | 3.4464e-05 | Intergenic | *MOGAT2*, ENSBTAG00000053794, ENSBTAG00000047111, ENSBTAG00000017443, ENSBTAG00000015091, ENSBTAG00000052936 | 1 |
|  | 60,972,100 | 9.2135e-06 | Upstream | ***FSHB***, *ARL14EP* | 2 |
| BTA16 | 76,071,433 | 5.7283e-06 | Intron | *CFHR5*, ***ASPM***, *ZBTB41* | 1 |
| BTA17 | 32,848,516 | 3.9645e-05 | Intergenic | None | 0 |
|  | 73,040,719 | 3.5788e-05 | Intergenic | *TANGO2*, ENSBTAG00000049742*, *DGCR8*, *TRMT2A*, ENSBTAG00000049062, *RANBP1*, *ZDHHC8*, *CCDC188*, ENSBTAG00000052630*, *RTN4R*, *PRODH*, *DGCR6L* | 8 |
| BTA18 | 35,987,322 | 1.0126e-05 | Intron | *ZFP90*, ENSBTAG00000053442, ***CDH3***, *CDH1* | 0 |
|  | 39,797,494 | 1.7387e-05 | Intergenic | *CALB2*, *CMTR2*, ENSBTAG00000051512 | 0 |
|  | 42,764,076 | 4.0823e-05 | Intergenic | *ZNF507* | 1 |
| BTA19 | 59,744,349 | 2.5462e-06 | Intergenic | None | 0 |
| BTA20 | 6,794,597 | 2.6598e-05 | Intron | *FAM169A*, *NSA2*, ***GFM2***, *HEXB*, ENSBTAG00000034138 | 0 |
|  | 15,788,934 | 1.0817e-05 | Intergenic | None | 0 |
|  | 52,149,342 | 2.6386e-05 | Intergenic | None | 0 |
|  | 71,002,191 | 3.7111e-05 | Upstream | ENSBTAG00000051832*, ***LPCAT1***, *SLC6A3* | 0 |
| BTA21 | 11,611,458 | 1.0837e-05 | Intergenic | ENSBTAG00000049211* | 0 |
|  | 16,945,728 | 1.1609e-05 | Intergenic | *AGBL1* | 0 |
|  | 16,961,979 | 2.4541e-05 | Intergenic |  |  |
| BTA22 | 5,160,840 | 3.4229e-06 | Intron | ***TGFBR2***, *GADL1* | 1 |
|  | 44,200,445 | 2.7232e-05 | Intron | ***ARHGEF3*** | 0 |
|  | 56,807,677 | 6.4738e-06 | Intron | ***PPARG***, ENSBTAG00000052393* | 0 |
|  | 57,243,658 | 8.9994e-06 | Intron | ENSBTAG00000031115, *RBSN*, ***MRPS25***, *NR2C2* | 2 |
| BTA23 | 1,002,264 | 5.7615e-05 | Intergenic | *KHDRBS2* | 0 |
|  | 2,330,898 | 1.4779e-06 | Intergenic | None | 0 |
|  | 2,804,180 | 5.7963e-05 | Intron | ***PRIM2*** | 2 |
|  | 2,823,702 | 2.9756e-05 | Intron |  |  |
|  | 3,504,801 | 2.3426e-05 | Intron | **ENSBTAG00000051082** | 0 |
|  | 17,164,665 | 4.2936e-05 | Downstream | *POLR1C*, *XPO5*, *POLH*, *GTPBP2*, ***MAD2L1BP***, ***RSPH9***, *MRPS18A*, ENSBTAG00000050989* | 5 |
|  | 18,203,819 | 4.6109e-05 | Intergenic | *SUPT3H* | 0 |
|  | 28,726,064 | 4.2777e-05 | Upstream | *JSP.1*, ENSBTAG00000037421, ***BOLA***, *TRIM26*, *TRIM15*, *TRIM10* | 0 |
| BTA25 | 8,702,138 | 1.1924e-05 | Intergenic | *GRIN2A* | 0 |
|  | 38,925,176 | 6.275e-05 | Coding | *FBXL18*, ENSBTAG00000050734*, ***TNRC18***, *SLC29A4*, *WIPI2* | 3 |
|  | 40,356,262 | 2.4382e-05 | Intergenic | *CARD11* | 0 |
|  | 40,856,888 | 1.7358e-05 | Intergenic | *LFNG*, *GRIFIN*, *CHST12*, ENSBTAG00000038978, *EIF3B*, *SNX8* | 1 |
|  | 41,219,195 | 2.1175e-05 | Intron | ***MAD1L1***, *ELFN1* | 0 |
| BTA26 | 10,927,671 | 2.6221e-05 | Intergenic | ENSBTAG00000054811*, *LIPA* | 1 |
|  | 11,356,804 | 2.3581e-05 | Intron | *PANK1*, ***KIF20B*** | 0 |
| BTA27 | 24,754,025 | 3.2687e-05 | Intron | *CLDN23*, **ENSBTAG00000053191** | 1 |
|  | 43,998,949 | 5.342e-06 | Intergenic | *ZNF385D* | 1 |
| BTA28 | 24,802,568 | 2.0176e-05 | Intron | *PBLD*, *HNRNPH3*, ***RUFY2***, *DNA2*, *SLC25A16* | 0 |
|  | 29,298,316 | 2.4334e-05 | Intron | NUDT13, *ECD*, *FAM149B1*, *DNAJC9*, ***MRPS16***, *CFAP70*, *ANXA7* | 0 |
|  | 37,896,374 | 3.454e-05 | Intron | ***NRG3*** | 1 |
|  | 40,034,955 | 5.309e-06 | Intergenic | None | 0 |
| BTA29 | 3,939,059 | 1.0245e-05 | Intergenic | None | 0 |
|  | 13,401,407 | 4.9536e-05 | Intergenic | None | 1 |

^1^ Candidate genes are represented by gene symbol when available, otherwise by the Ensembl gene ID. The long noncoding RNA genes are marked by asterisk (*). The genes directly linked to SNPs are further denoted in bold.

^2^ The number of known and reproduction-associated QTL found in Cattle QTL Database v43 (www.animalgenome.org).

**Table S11. The previously reported and reproduction-associated QTL for the significant SNPs revealed by multiple-trait analysis of 18 heifer, cow, and sire traits**

| **SNPs** | | **Known QTL** | |
| --- | --- | --- | --- |
| **Chromosome** | **Position bp** | **ID** | **Name** |
| BTA5 | 9837342 | 24589 | Calving ease |
| BTA6 | 93858468 | 173429 | Daughter pregnancy rate |
|  | 93858468 | 173430 | Daughter pregnancy rate |
|  | 93858468 | 176402 | Conception rate |
|  | 93858468 | 176403 | Conception rate |
|  | 93858468 | 176404 | Conception rate |
| BTA7 | 96545772 | 107103 | Conception rate |
|  | 96545772 | 107104 | Conception rate |
|  | 96545772 | 30040 | Interval to first estrus after calving |
|  | 96545772 | 30324 | Interval to first estrus after calving |
|  | 100189059 | 29910 | Age at puberty |
|  | 100189059 | 29905 | Age at puberty |
| BTA8 | 60191474 | 212644 | Inseminations per conception |
|  | 85035847 | 107113 | Conception rate |
|  | 85035847 | 43714 | Calving ease maternal |
|  | 85035847 | 43716 | Daughter pregnancy rate |
|  | 85035847 | 43725 | Calving ease |
|  | 85035847 | 43727 | Stillbirth |
| BTA10 | 15091095 | 212225 | First service conception |
|  | 15091095 | 212688 | Inseminations per conception |
|  | 39839364 | 176768 | First service conception |
|  | 43187675 | 181267 | Conception rate |
|  | 43187675 | 181492 | Inseminations per conception |
| BTA12 | 51864428 | 46434 | Calving ease maternal |
|  | 51864428 | 46436 | Daughter pregnancy rate |
|  | 51864428 | 46448 | Calving ease |
|  | 51864428 | 46449 | Stillbirth |
| BTA13 | 35506150 | 212762 | Inseminations per conception |
|  | 35506150 | 30085 | Interval to first estrus after calving |
|  | 35506150 | 30294 | Interval to first estrus after calving |
| BTA15 | 54978763 | 107149 | Conception rate |
|  | 60972100 | 212802 | Inseminations per conception |
|  | 60972100 | 212292 | First service conception |
| BTA16 | 76071433 | 177190 | Conception rate |
| BTA17 | 73040719 | 181526 | Inseminations per conception |
|  | 73040719 | 181354 | Conception rate |
|  | 73040719 | 177211 | Conception rate |
|  | 73040719 | 176830 | First service conception |
|  | 73040719 | 140628 | Non-return rate |
|  | 73040719 | 142307 | Non-return rate |
|  | 73040719 | 122410 | Conception rate |
|  | 73040719 | 57101 | Daughter pregnancy rate |
| BTA18 | 42764076 | 125245 | Twinning |
| BTA22 | 5160840 | 121667 | Calving interval |
|  | 57243658 | 107215 | Conception rate |
|  | 57243658 | 107038 | Daughter pregnancy rate |
| BTA23 | 2804180 | 51892 | Stillbirth maternal |
|  | 2804180 | 51903 | Stillbirth maternal |
|  | 2823702 | 51892 | Stillbirth maternal |
|  | 2823702 | 51903 | Stillbirth maternal |
|  | 17164665 | 212937 | Inseminations per conception |
|  | 17164665 | 176853 | First service conception |
|  | 17164665 | 177264 | Conception rate |
|  | 17164665 | 52095 | Calving ease maternal |
|  | 17164665 | 52102 | Calving ease |
| BTA25 | 38925176 | 121698 | Calving interval |
|  | 38925176 | 52564 | Daughter pregnancy rate |
|  | 38925176 | 52569 | Calving ease |
|  | 40856888 | 177281 | Conception rate |
| BTA26 | 10927671 | 29950 | Age at puberty |
| BTA27 | 24754025 | 62078 | Sperm motility |
|  | 43998949 | 121670 | Calving interval |
| BTA28 | 37896374 | 53812 | Calving ease |
| BTA 29 | 13401407 | 181417 | Conception rate |


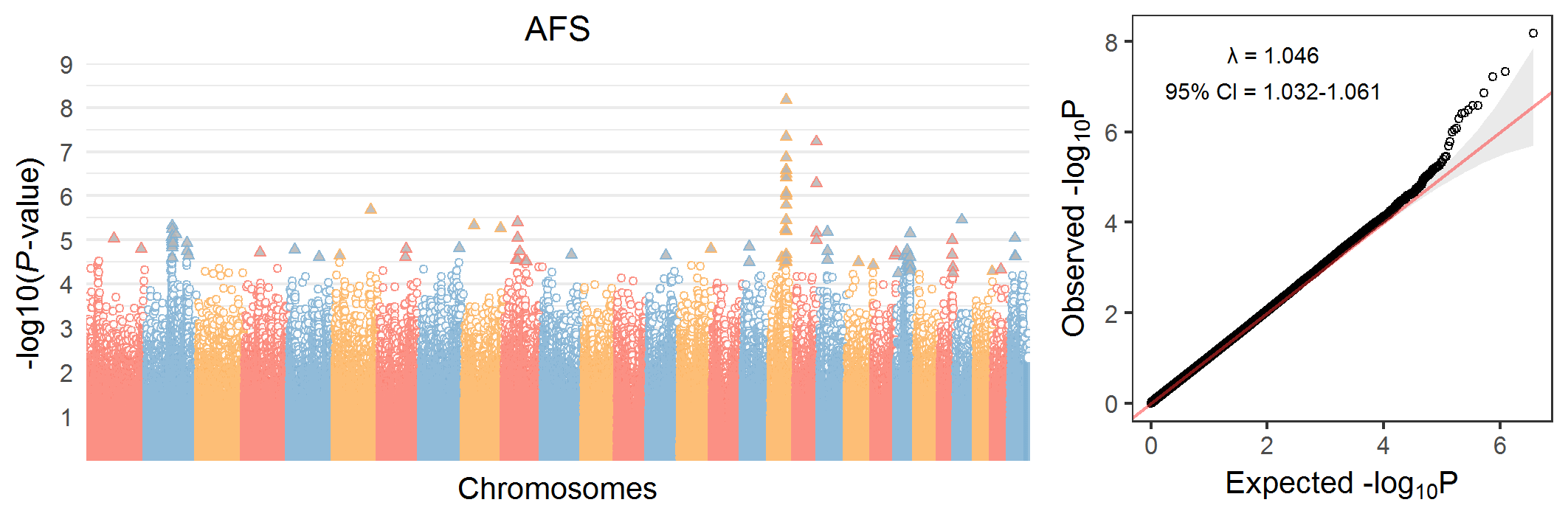


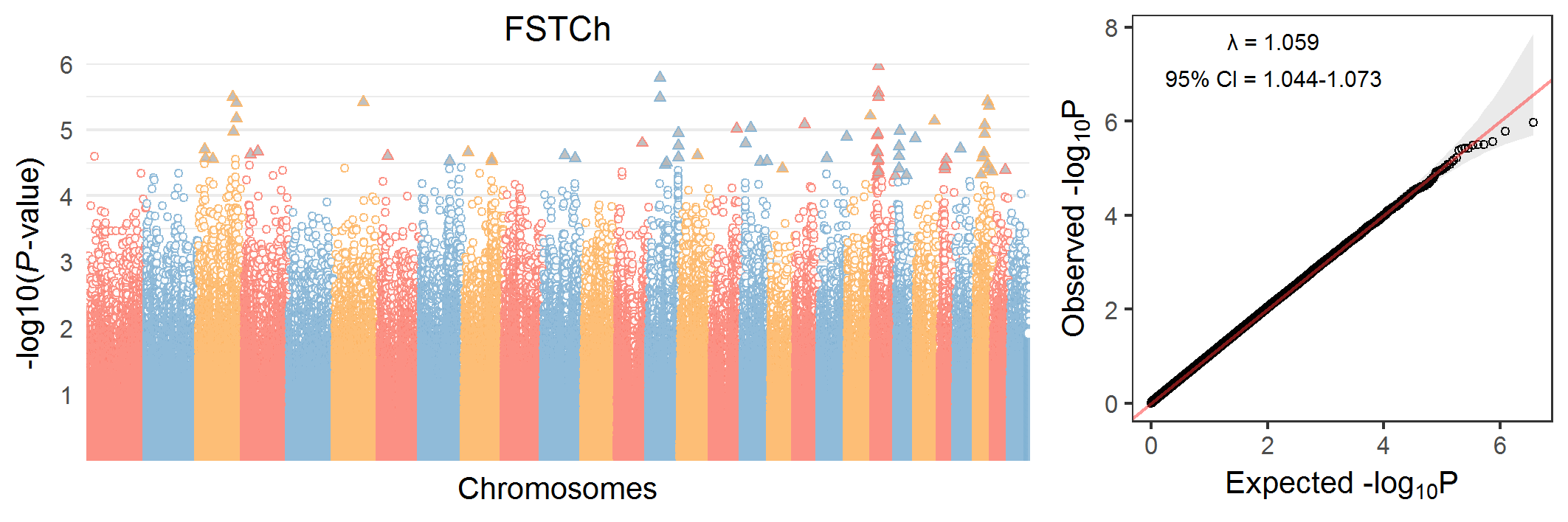


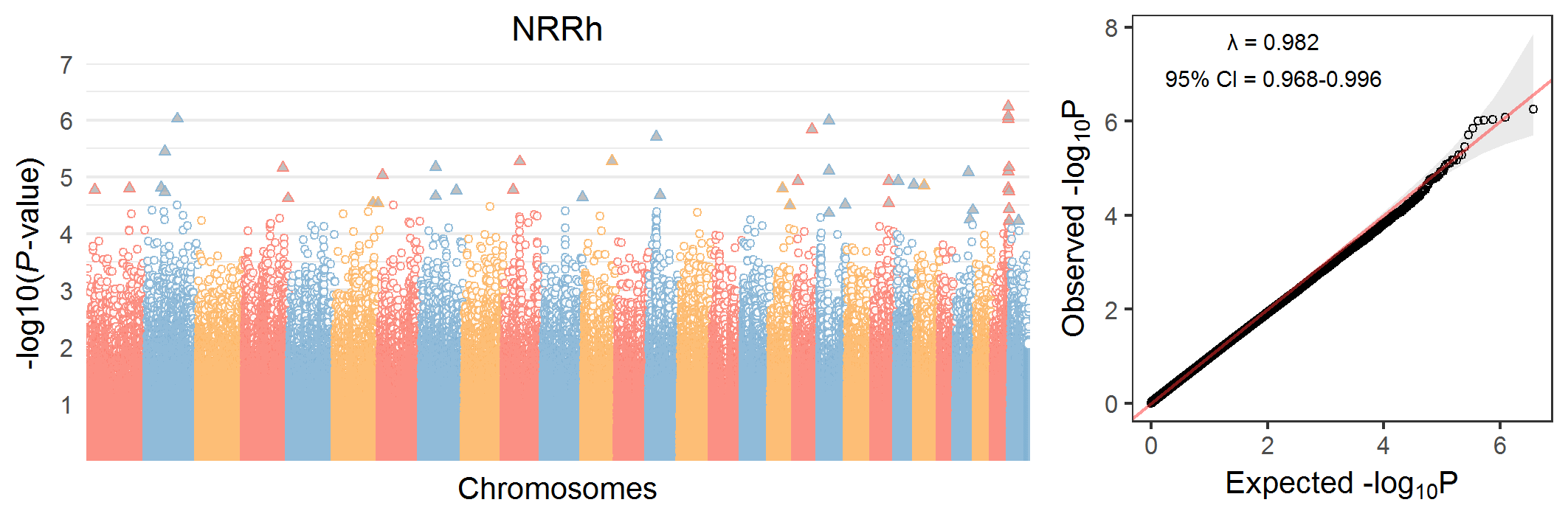


**Fig. S1.** **Manhattan plots (left) and Quantile-quantile plots (right) of the single-trait GWAS results based on imputed whole-genome sequence data**. Statistically significant SNP are denoted by triangles. AFS = Age at first service; FSTCh = First service to conception heifer; NRRh = Non-return rate heifer; CEh = Calving ease heifer; CSh = Calf survival heifer; CA = Calving ability; CTFS =Calving to first service; DO = Days open; FSTCc = First service to conception cow; NRRc = Non-return rate cow; CEc = Calving ease cow; CSc = Calf survival cow; DCA = Daughter calving ability; DF = Daughter fertility; SCEh = Sire calving ease heifer; SCEc = Sire calving ease cow; SCSh = Sire calf survival heifer; SCSc = Sire calf survival cow; MAF = minor allele frequencies; λ = genomic inflation factor; CI = confidence interval.


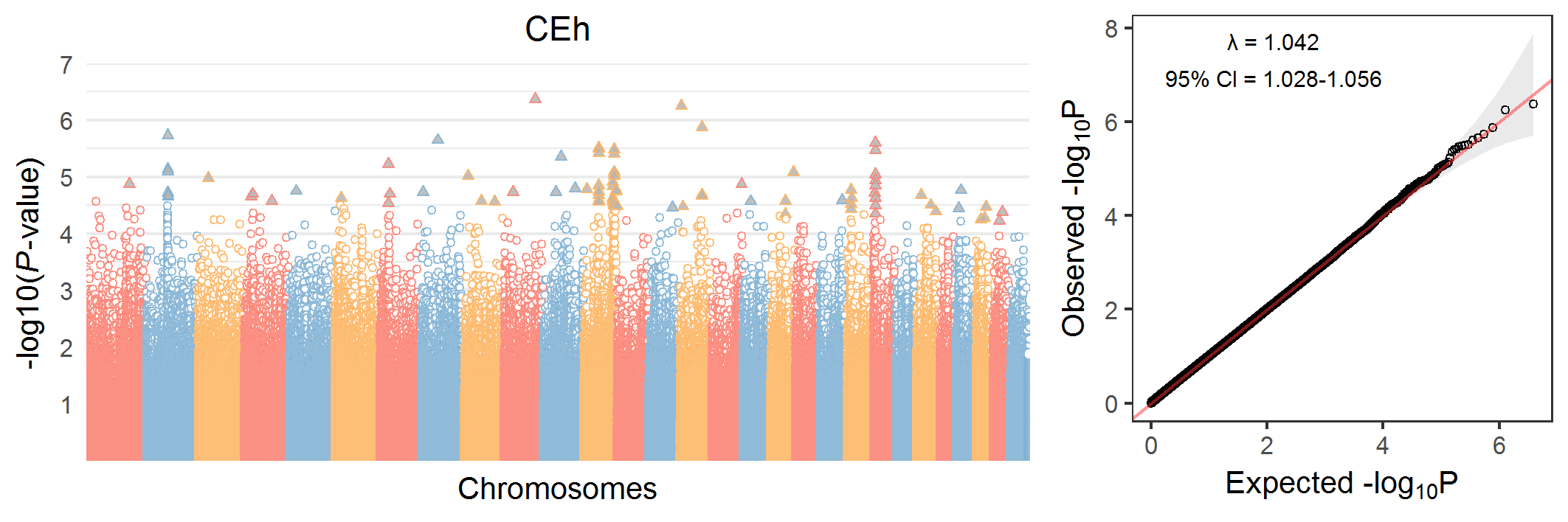


**
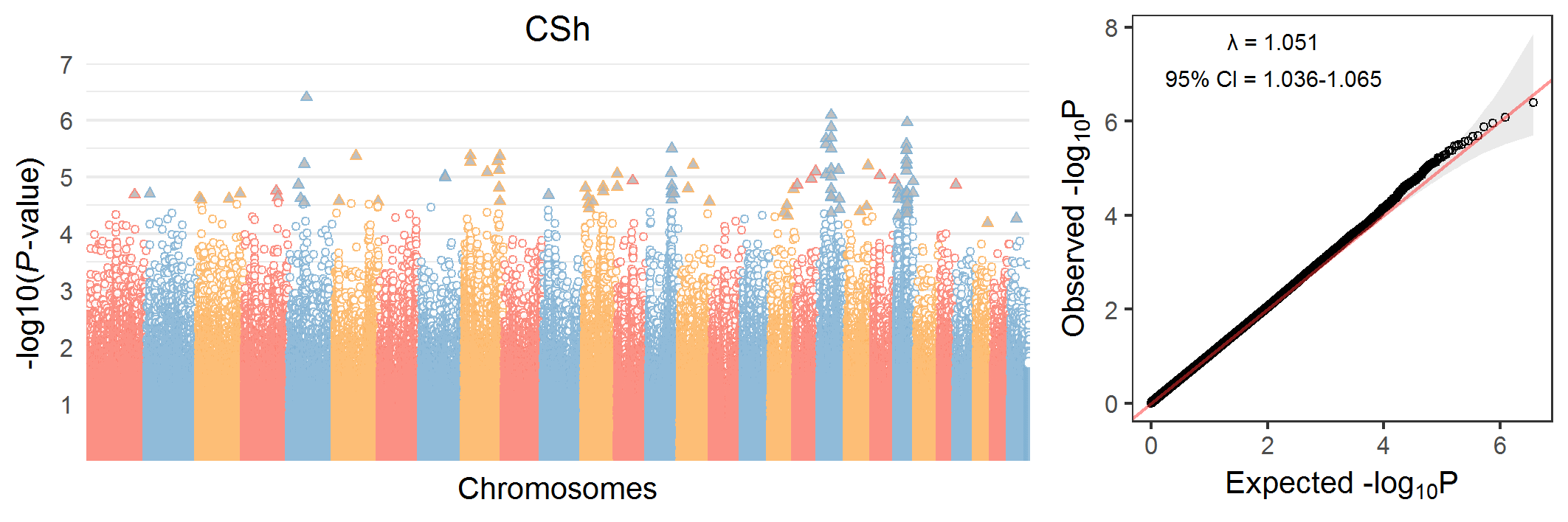
**

**
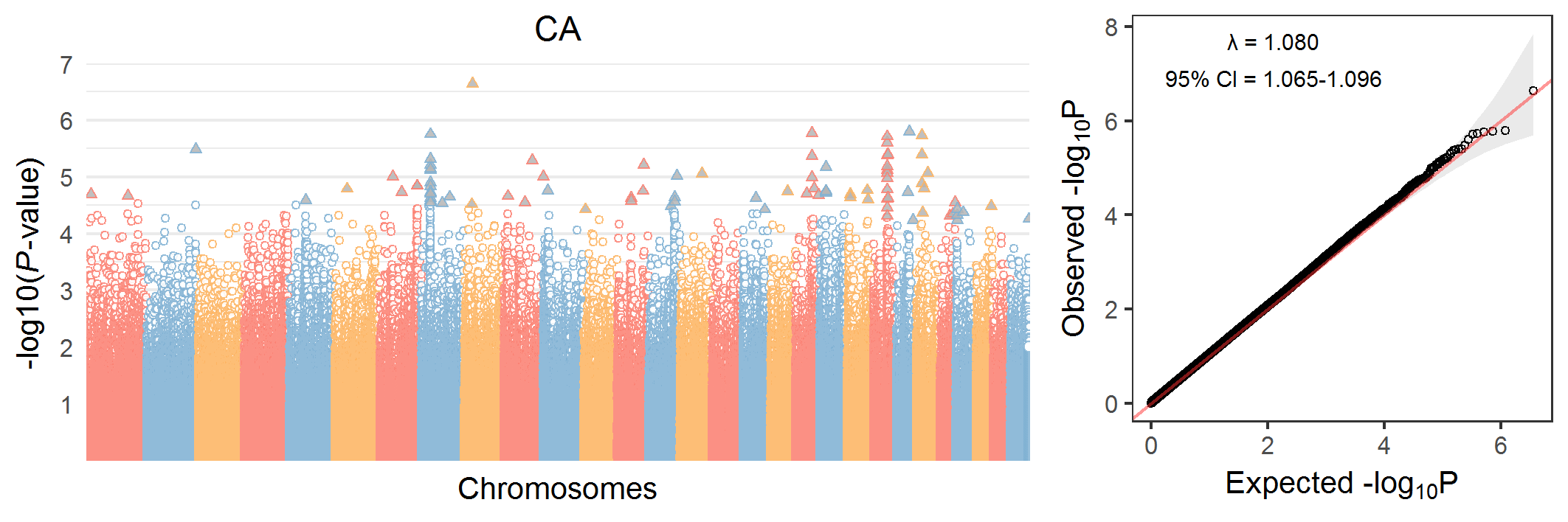
**

**
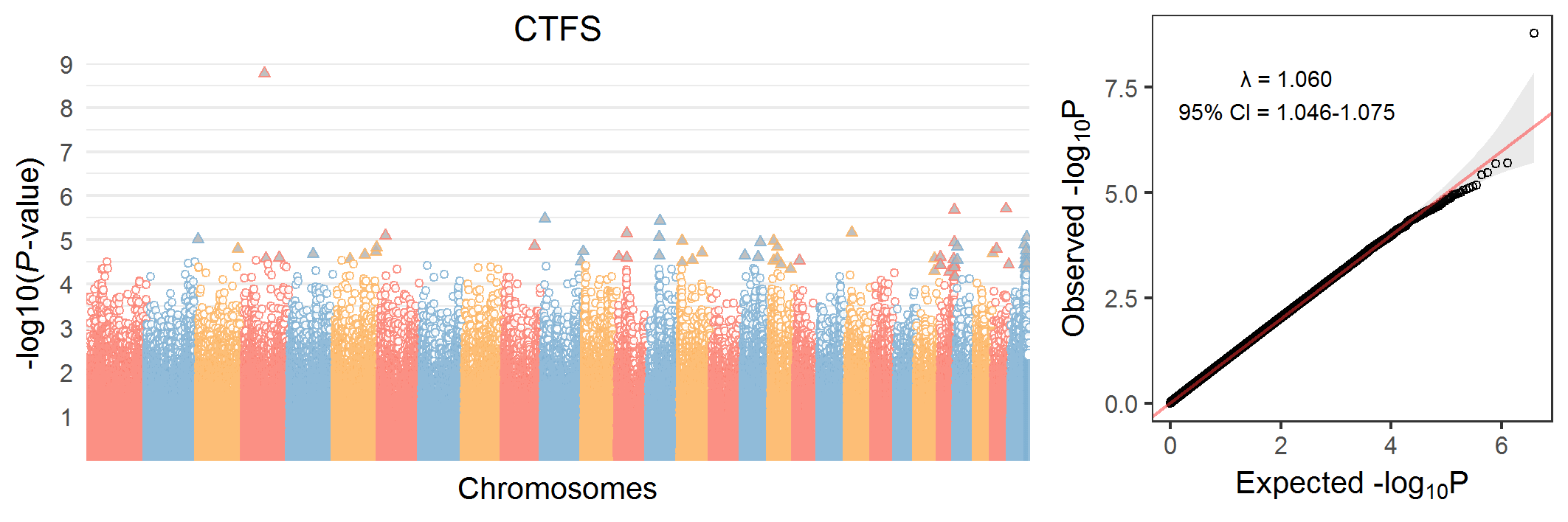
**

**Fig. S1 continued**

**
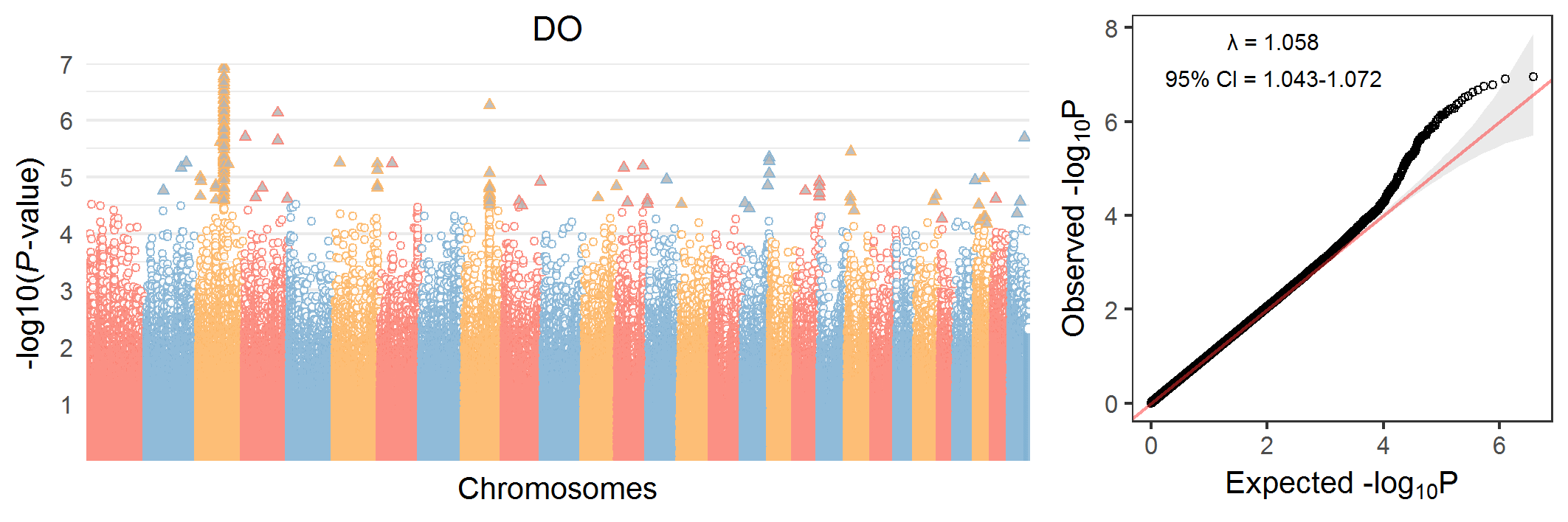
**

**
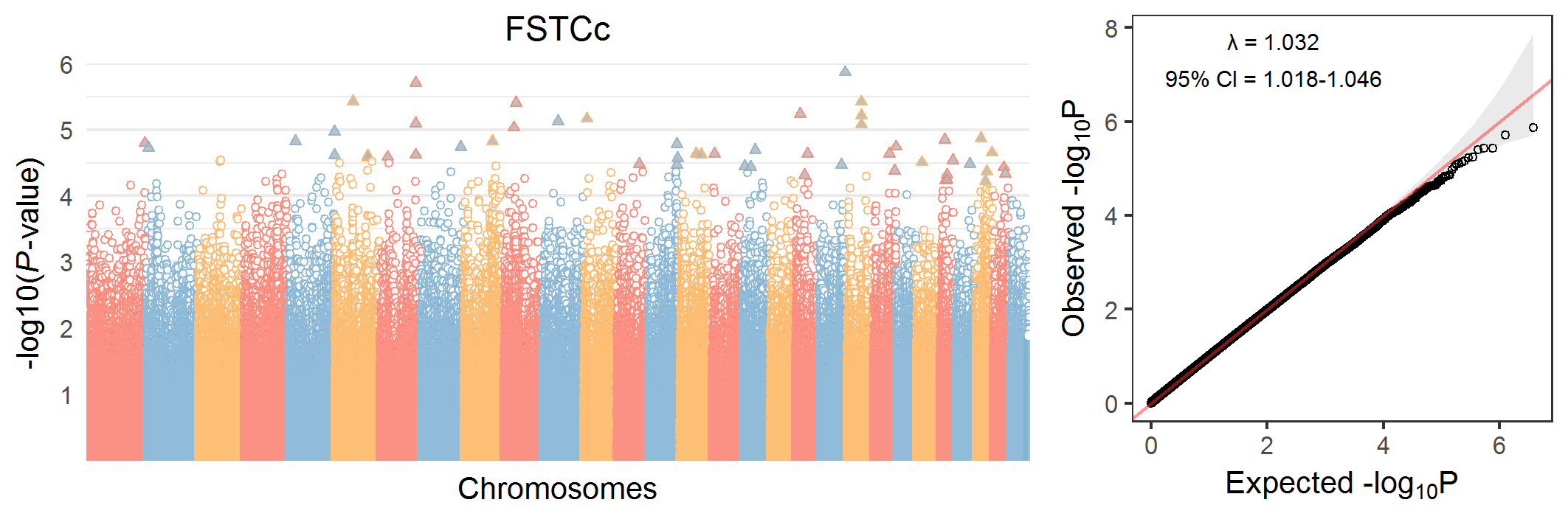
**

**
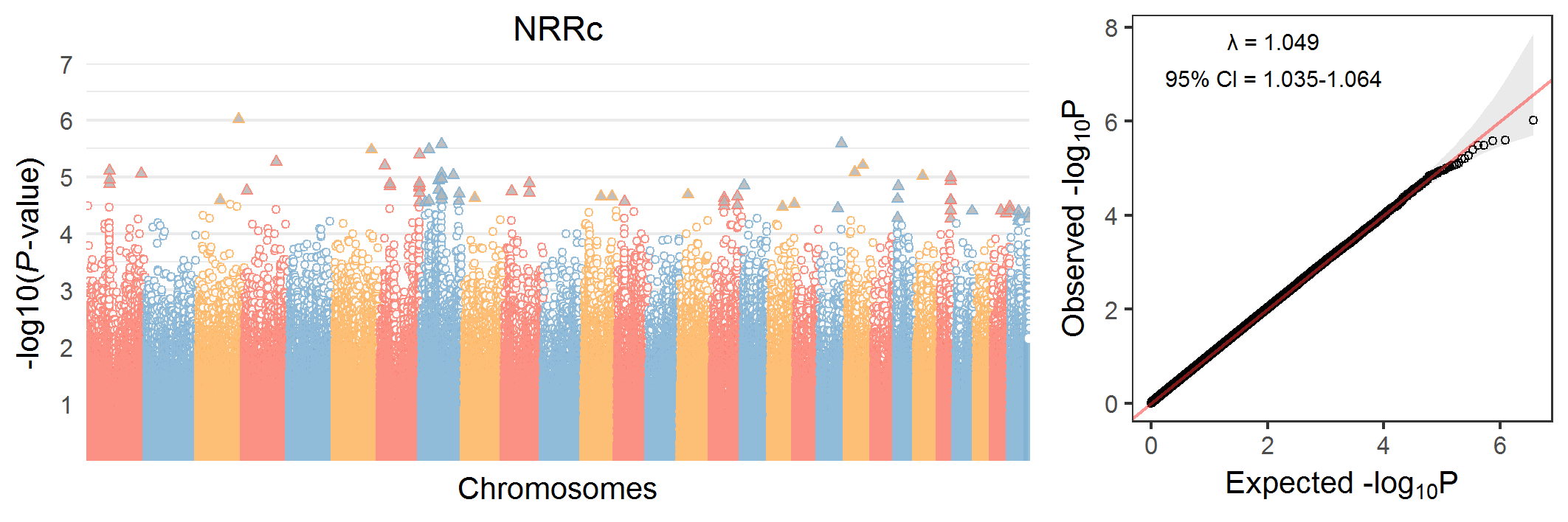
**

**
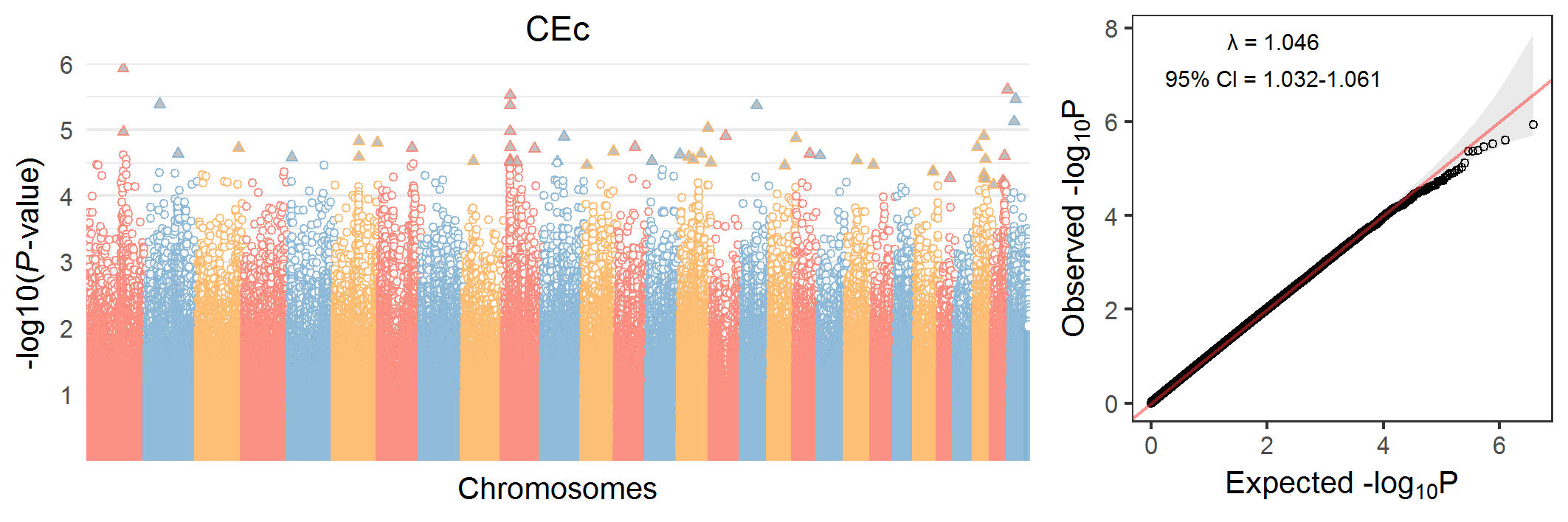
**

**Figure S1 continued**

**
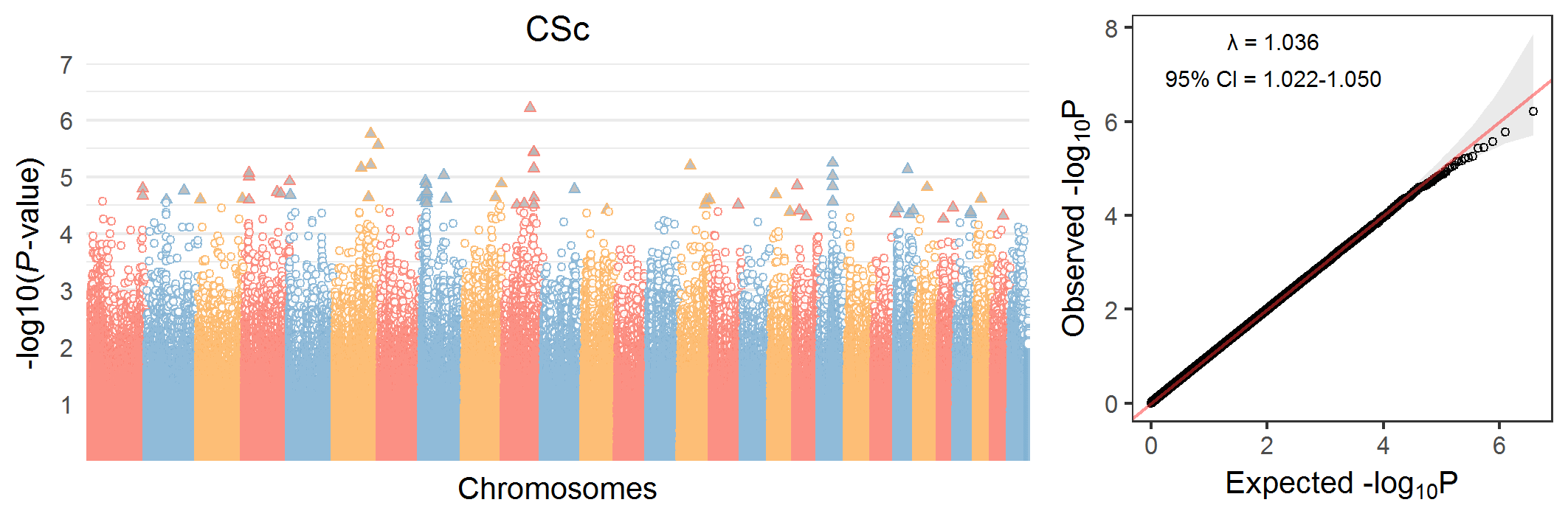
**

**
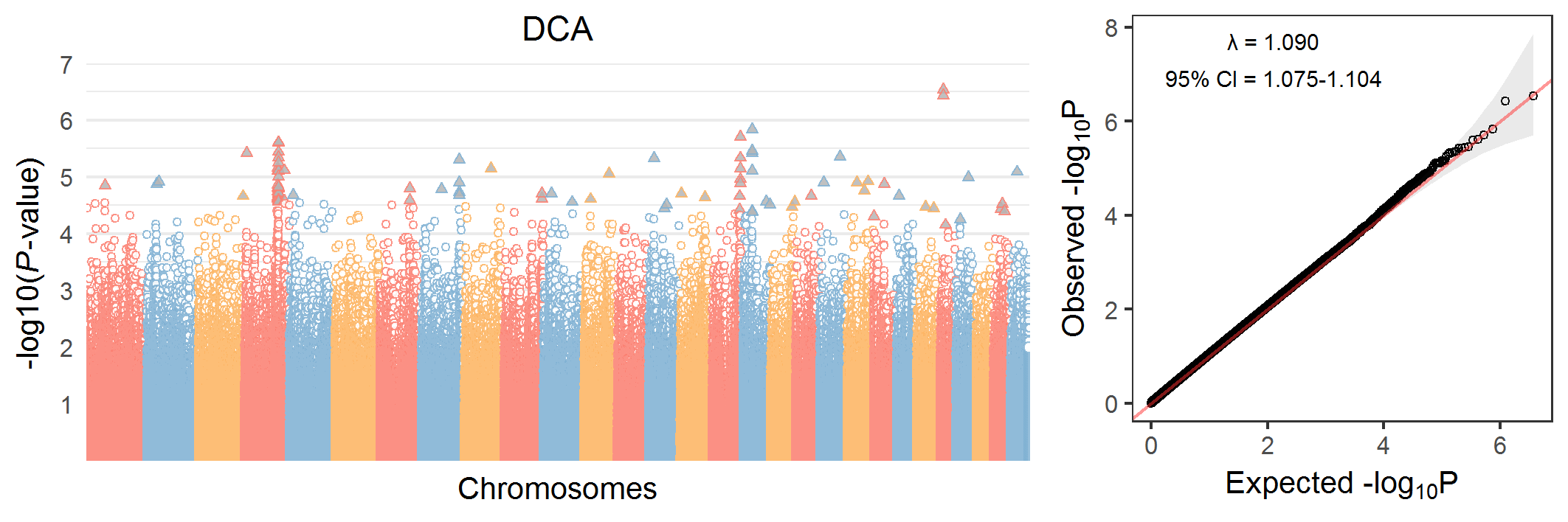
**

**
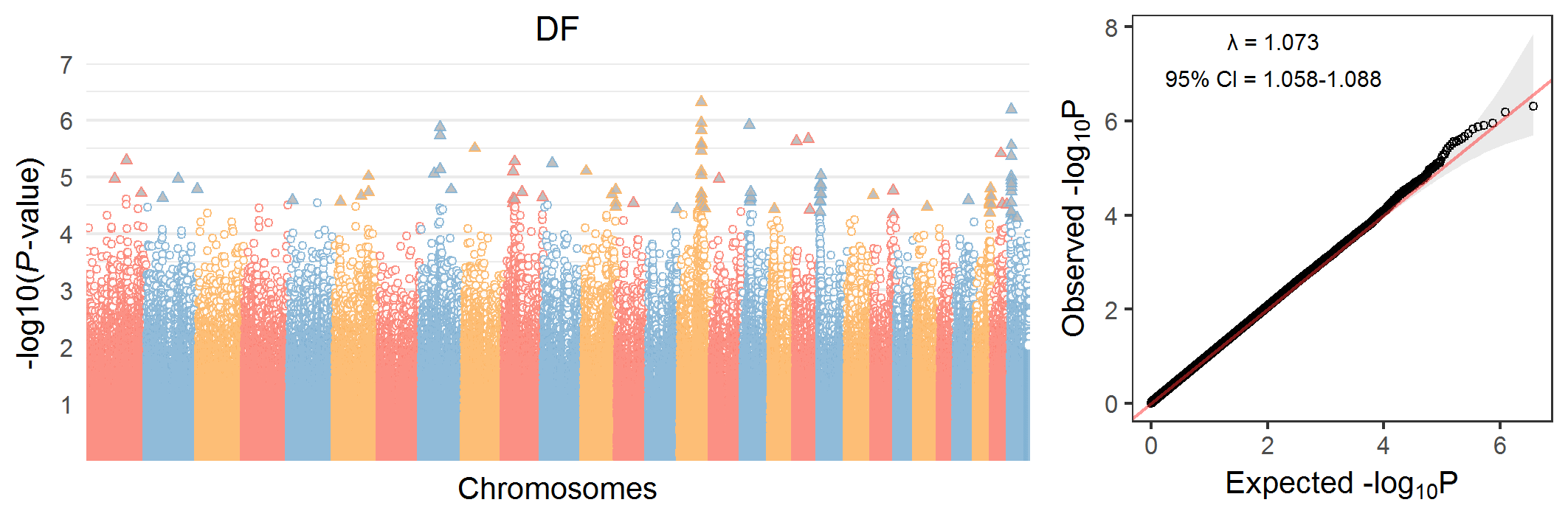
**

**
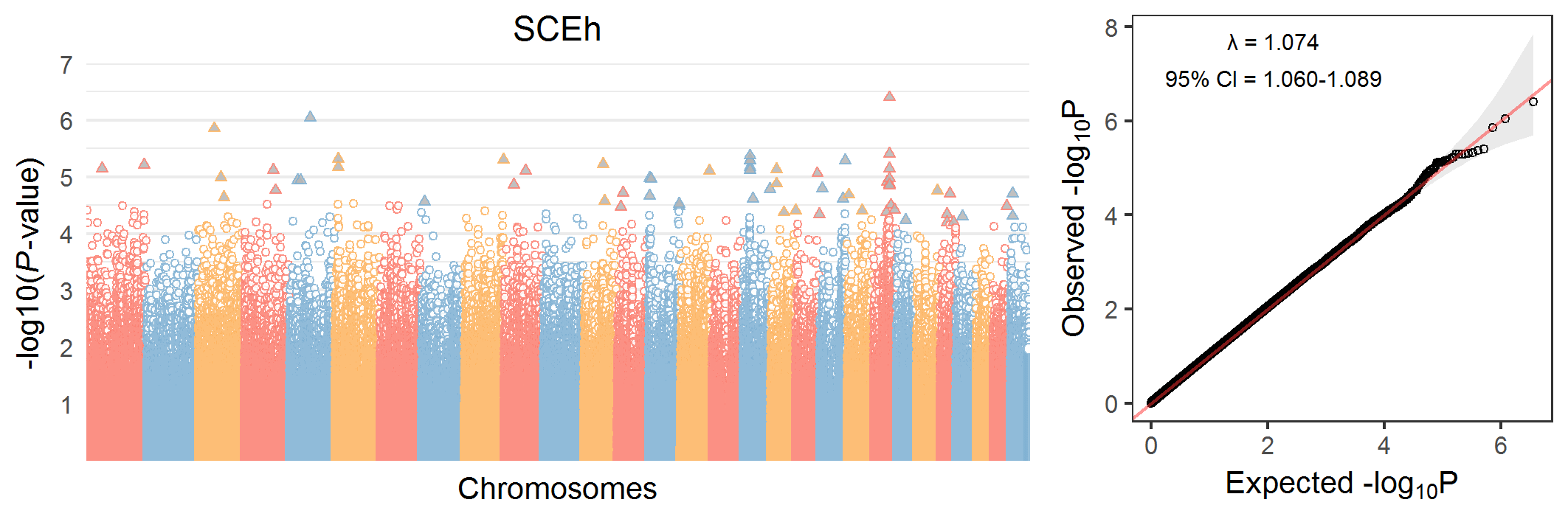
**

**Figure S1 continued**

**
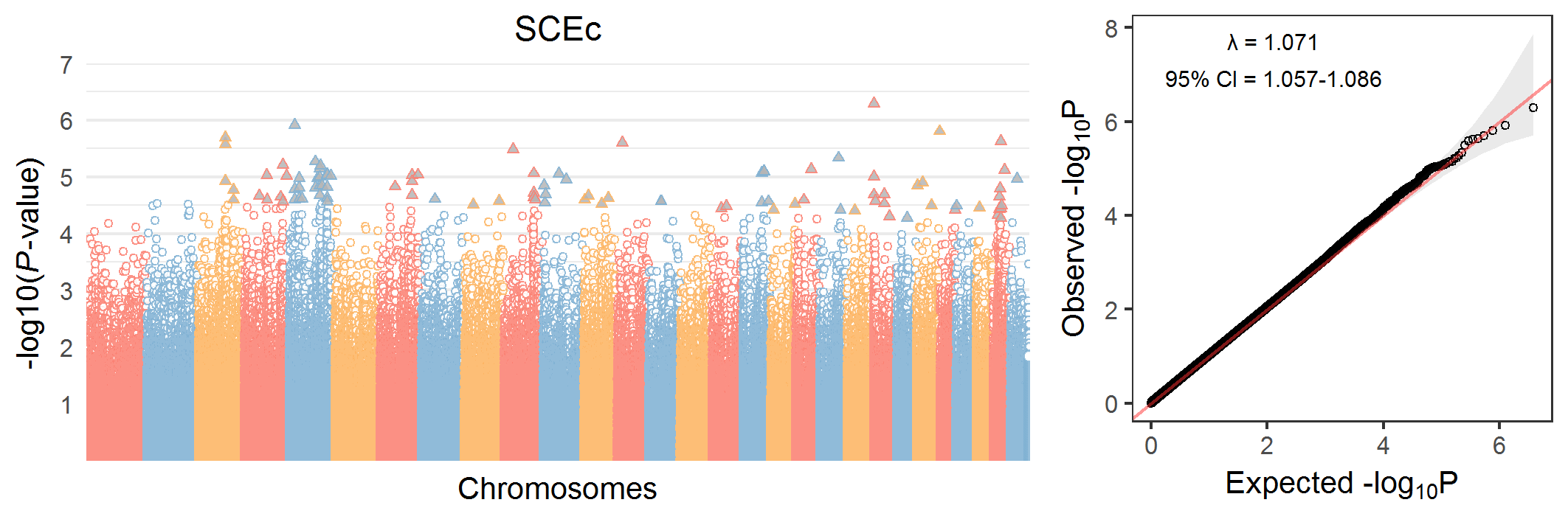
**

**
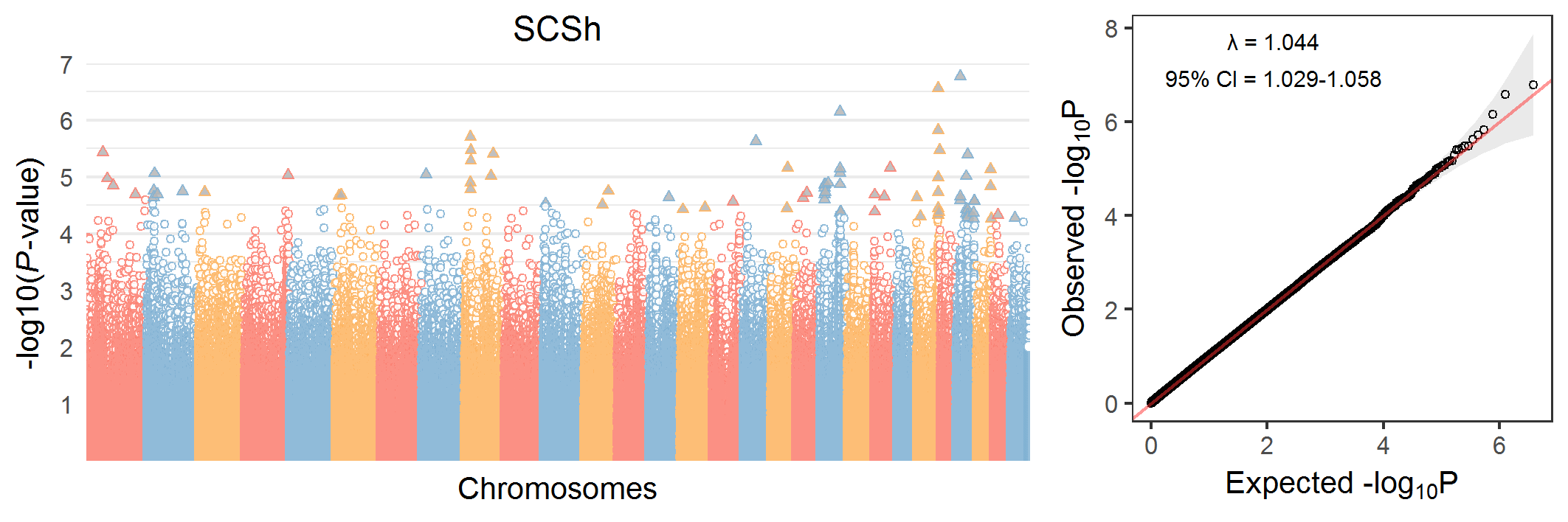
**

**
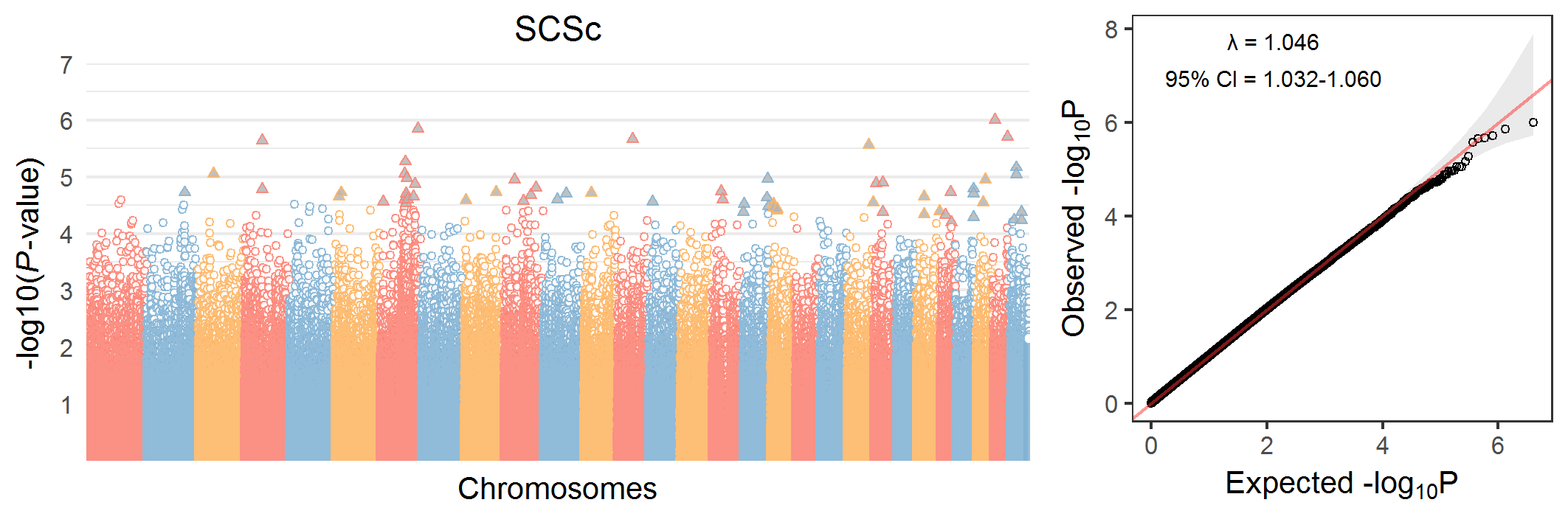
**

**Figure S1 continued**

**
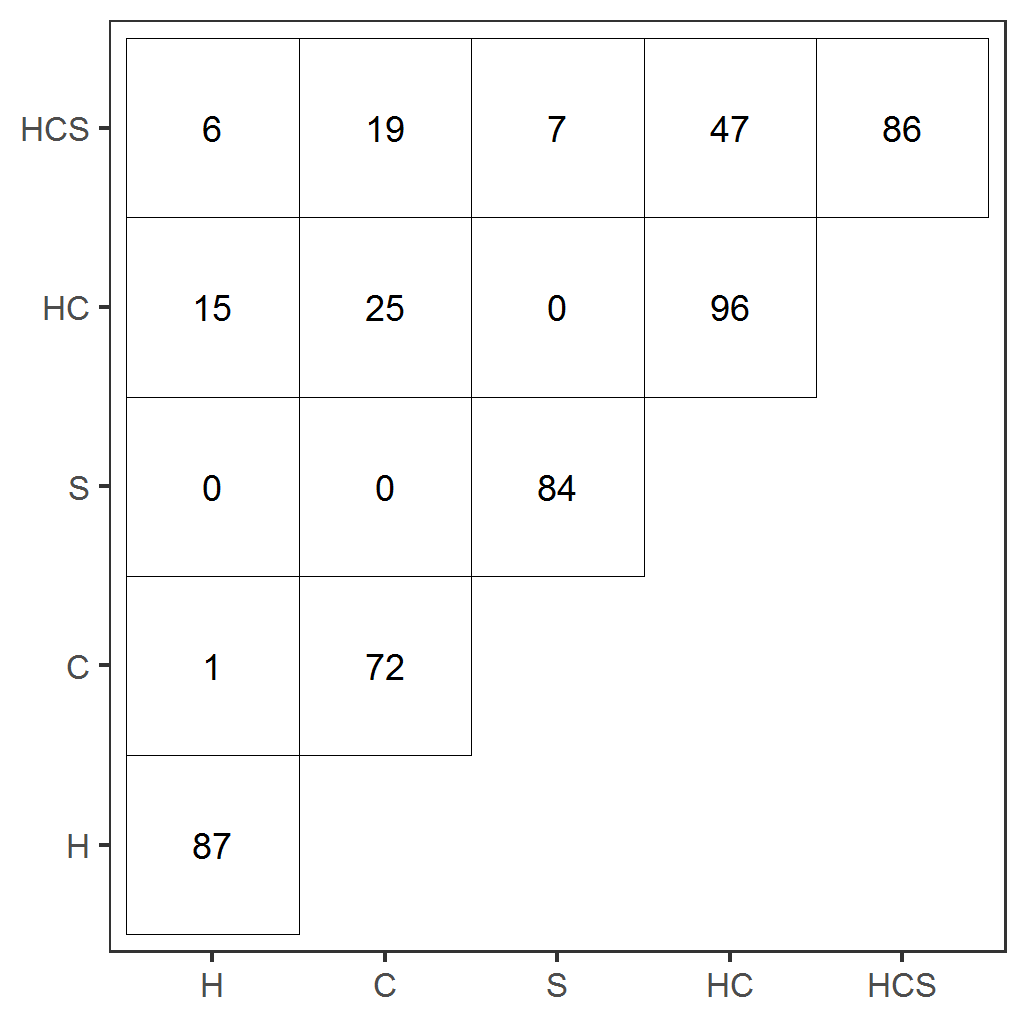
**

**Fig. S2.** **Numbers of significant SNPs for each trait category and their overlaps found by multiple-trait analysis**. H = Heifers; C = Cows; S = Sires; HC = Heifers plus Cows; HCS = all the animals together.


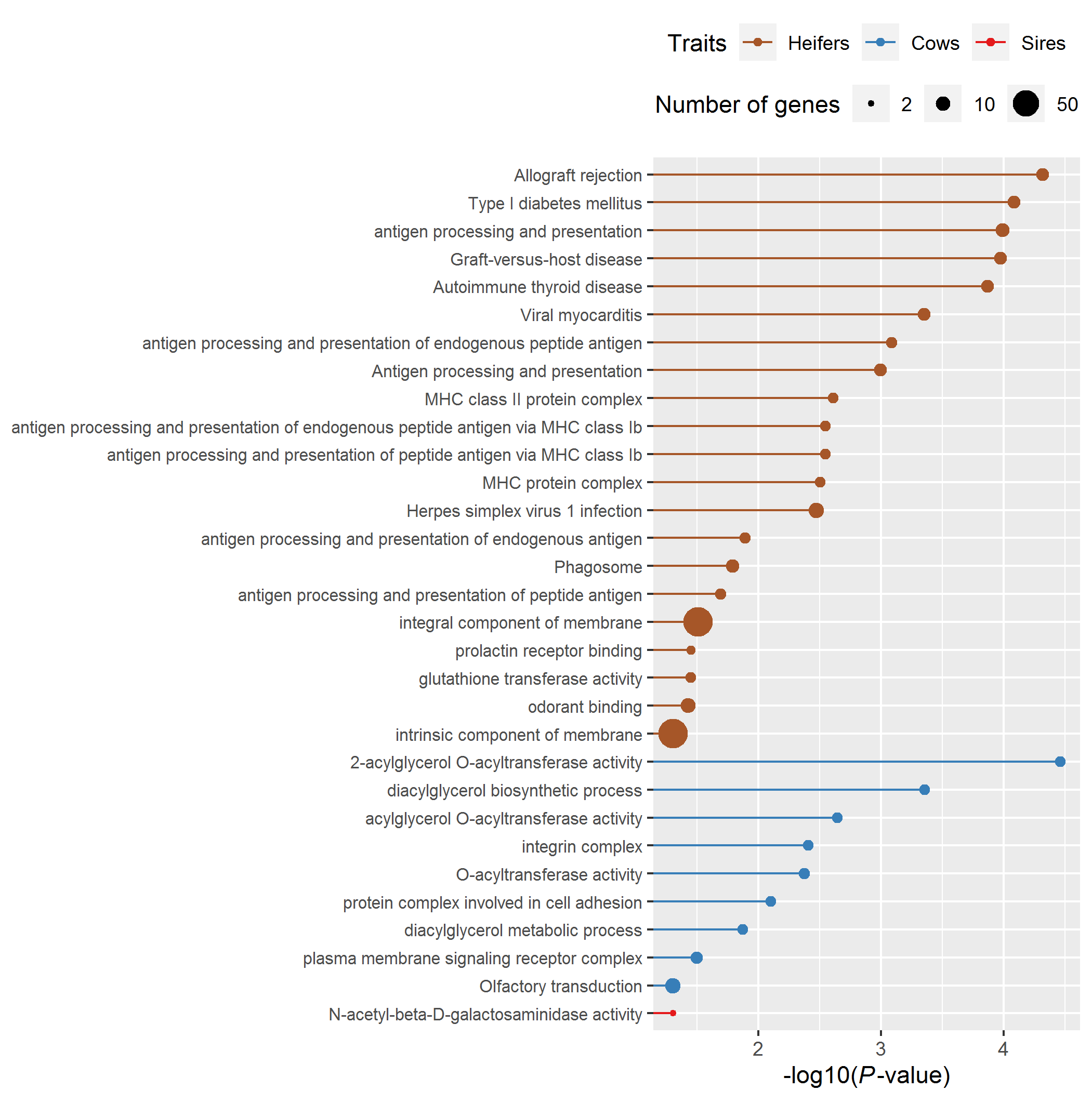


**Fig. S3. Significantly enriched biological functions of candidate genes revealed by multiple-trait analysis**.


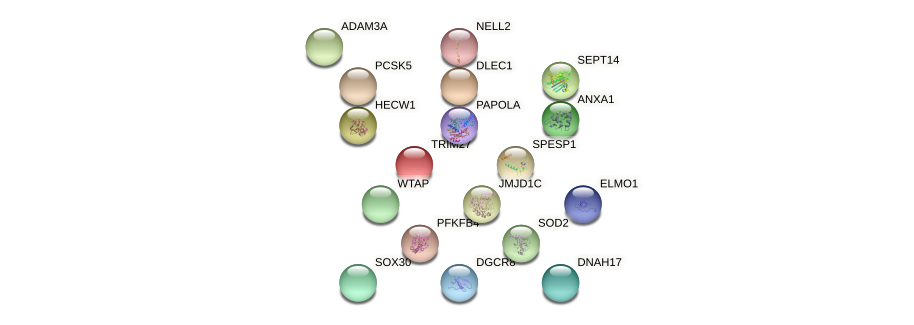


**Fig. S4.** Potential protein-protein interaction among biologically relevant genes identified for heifer, cow, and sire traits in this study.
